# Author Correction: The barley MLA13-AVR_A13_ heterodimer reveals principles for immunoreceptor recognition of RNase-like powdery mildew effectors

**DOI:** 10.1038/s44318-025-00456-7

**Published:** 2025-05-07

**Authors:** Aaron W Lawson, Andrea Flores-Ibarra, Yu Cao, Chunpeng An, Ulla Neumann, Monika Gunkel, Isabel M L Saur, Jijie Chai, Elmar Behrmann, Paul Schulze-Lefert

**Affiliations:** 1 Department of Plant Microbe Interactions, Max Planck Institute for Plant Breeding Research, 50829 Cologne, Germany; 2 University of Cologne, Faculty of Mathematics and Natural Sciences, Institute of Biochemistry, 50674 Cologne, Germany; 3 School of Life Sciences, Westlake University, 310031 Hangzhou, China; 4 Central Microscopy, Max Planck Institute for Plant Breeding Research, 50829 Cologne, Germany; 5 Institute for Plant Sciences, University of Cologne, 50674 Cologne, Germany; 6 Cluster of Excellence on Plant Sciences (CEPLAS), Max Planck Institute for Plant Breeding Research and University of Cologne, 50829 Cologne, Germany

## Abstract

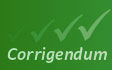

**Correction to:** *The EMBO Journal* (2025). 10.1038/s44318-025-00373-9 | Published online 13 February 2025

**The Author contributions are updated**.

The author contributions are updated from:

**Author contributions**

**Aaron W Lawson**: Conceptualisation; Data curation; Formal analysis; Validation; Investigation; Visualisation; Methodology; Writing—original draft; Project administration; Writing—review and editing. **Andrea Flores-Ibarra**: Formal analysis. **Yu Cao**: Investigation. **Chunpeng An**: Investigation. **Ulla Neumann**: Investigation. **Monika Gunkel**: Investigation. **Isabel M L Saur**: Investigation. **Jijie Chai**: Project administration. **Elmar Behrmann**: Project administration. **Paul Schulze-Lefert**: Project administration.

To:

**Aaron W Lawson**: Conceptualisation; Investigation; Data analysis; Writing; Editing. **Andrea Flores-Ibarra**: Structural model building. **Yu Cao**: Investigation. **Chunpeng An**: Investigation. **Ulla Neumann**: Electron microscopy screening. **Monika Gunkel**: Electron microscopy screening; data analysis. **Isabel M L Saur**: Investigation.

**Jijie Chai**: Conceptualisation; Project administration. **Elmar Behrmann**: Conceptualisation; Project administration; Data analysis; Structural model building; Writing; Editing. **Paul Schulze-Lefert**: Conceptualisation; Project administration; Writing; Editing.

