# Supplementary material for: The barley MLA13-AVRA13 heterodimer reveals principles for immunoreceptor recognition of RNase-like powdery mildew effectors
Source: EMBO J. 2025 Feb 13;44(11):3210–30. doi: 10.1038/s44318-025-00373-9 (PMC12130304; doi:10.1038/s44318-025-00373-9)
Supplement: Supplementary file 1 — Appendix [file 44318_2025_373_MOESM1_ESM.pdf]

Appendix for

The barley MLA13-AVR$_{A13}$ heterodimer reveals principles for immunoreceptor recognition of RNase-like powdery mildew effectors

Table of contents

Appendix Figure S1……………………………………………………………………2
Appendix Figure S2……………………………………………………………………3
Appendix Figure S3……………………………………………………………………4
Appendix Figure S4……………………………………………………………………5
Appendix Figure S5……………………………………………………………………6
Appendix Figure S6……………………………………………………………………7
Appendix Figure S7……………………………………………………………………8
Appendix Figure S8……………………………………………………………………9
Appendix Figure S9…………………………………………………………………..10
Appendix Table S1……………………………………………………………………11

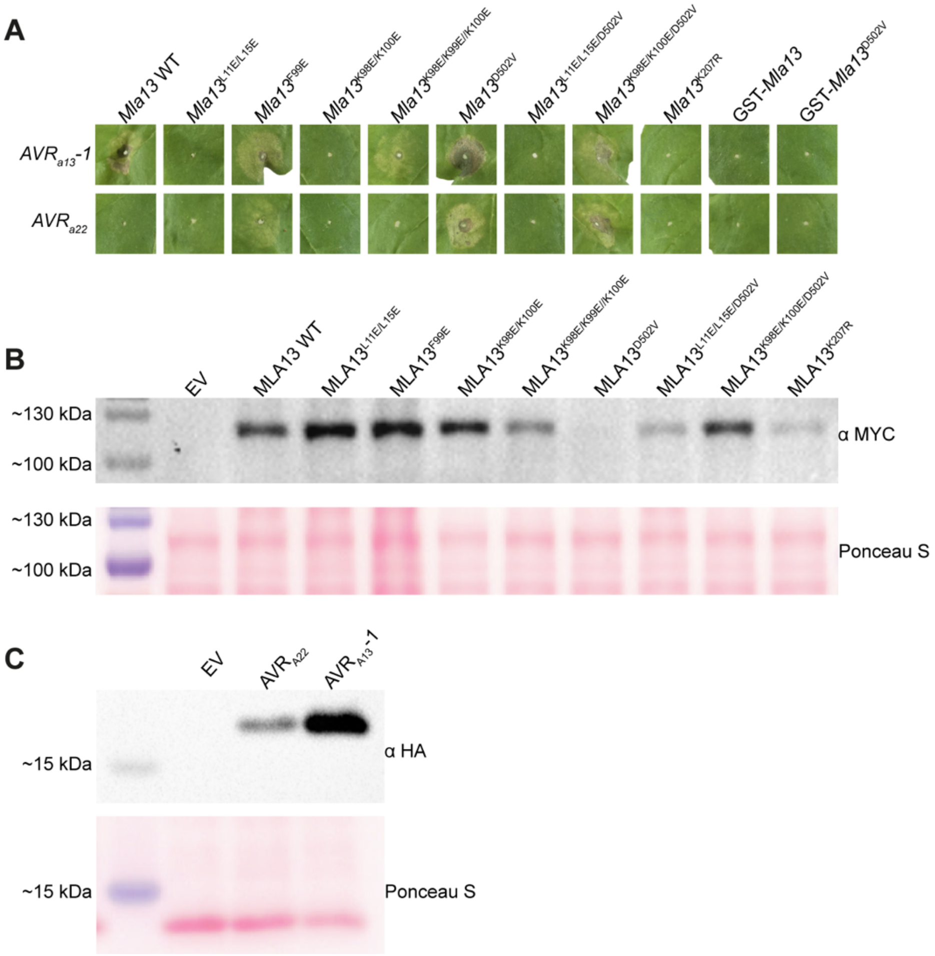

Appendix Figure S1. ***Agrobacterium*****-mediated co-expression of MLA13 variants with AVR$_{A13}$-1 and AVR$_{A22}$ in leaves of *N. benthamiana*.**

(A) Cell death phenotypes of MLA13 phenotypes of variants that result in effector-triggered HR, autoactive HR or loss of HR. Six independent replicates were performed (Source Data Figure 1). **(B)** Western blot analysis of the MLA13 variants in **(A)**. Samples were run on 10% SDS PAGE gels. **(C)** Western blot analysis of AVR$_{A13}$-1 and AVR$_{A22}$. Samples were run on 12% SDS PAGE gels.

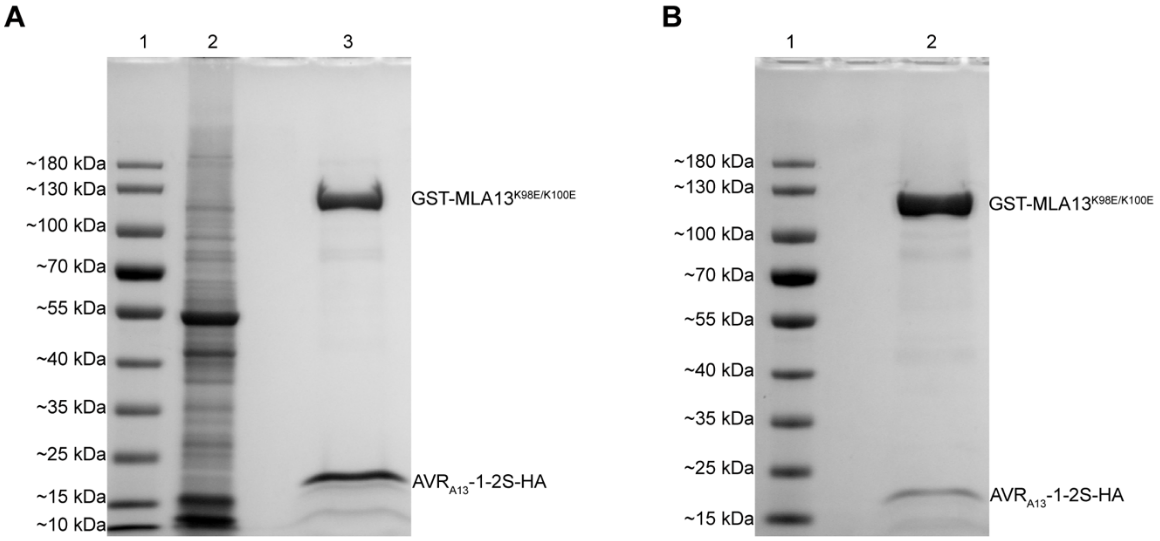

Appendix Figure S2. CBB-stained, SDS PAGE gel of the samples from a two-step affinity purification of the resolved MLA13$^{K98E/K100E}$-AVR$_{A13}$-1 heterodimer.

(A) Lane #1: ladder; lane #2: lysate (5 μL loaded); lane #3: first-step Twin-Strep eluate (45 μL/1 mL loaded). Samples run on a 10% gel. **(B)** Lane #1: ladder; lane #2: second-step GST eluate (45 μL/750 μL loaded). Samples run on a 10% gel.

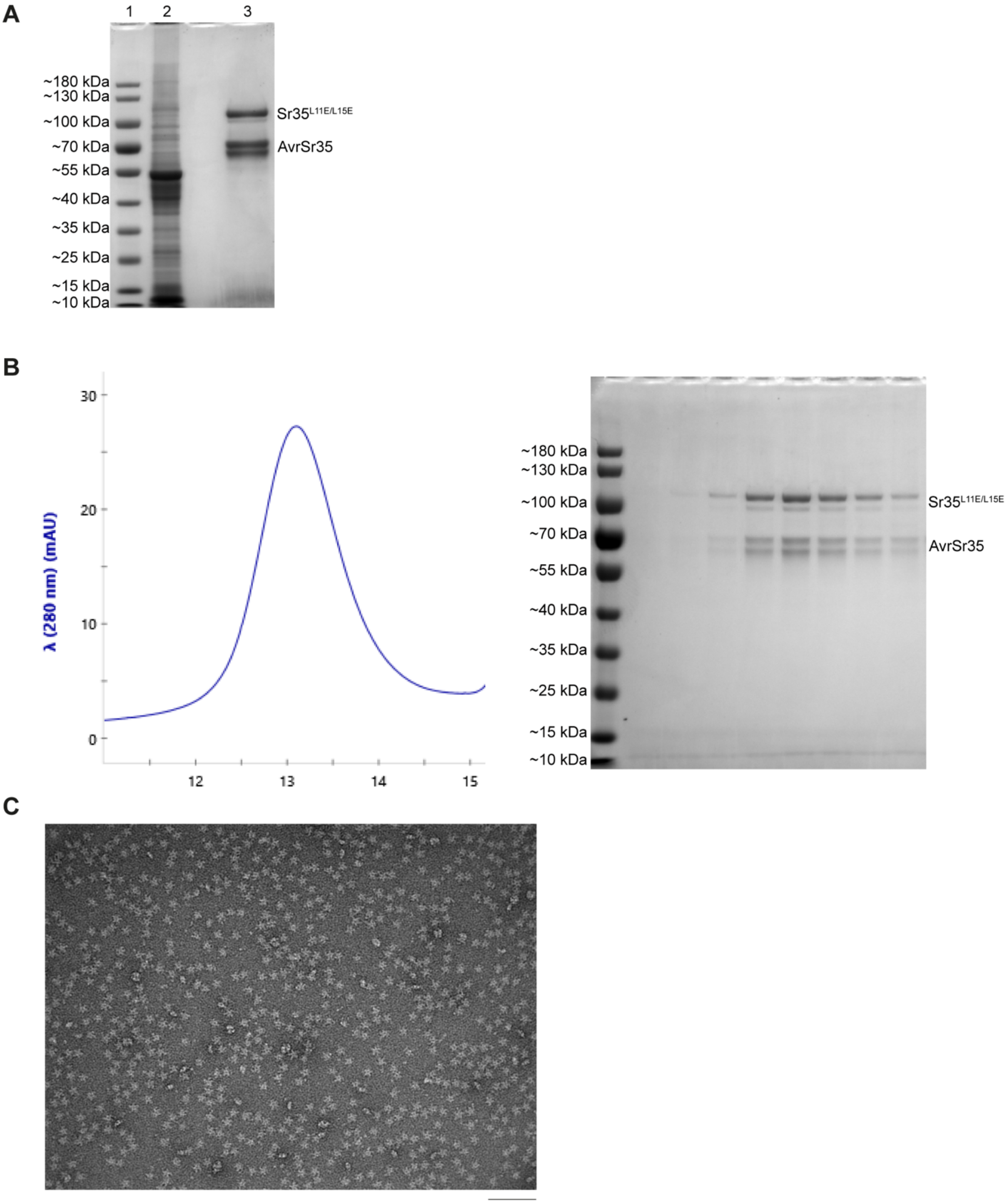

Appendix Figure S3. Transient expression and purification of the Sr35 resistosome from leaves of *N. benthamiana*.

(A) CBB-stained, SDS PAGE gel of a single step affinity purification of the Sr35$^{L11E/L15E}$ resistosome *via* the C-terminal, Twin-Strep-tag on AvrSr35. Lane #1: ladder; lane #2: lysate (5 µL loaded); lane #3: first-step eluate (45 µL/1 mL loaded). Samples run on a 10% gel. **(B)** SEC profile (left) and accompanying SDS PAGE gel of resulting elution fractions (right). Samples run on a 10% gel. **(C)** Negative staining TEM of a five-fold dilution from the fraction corresponding to the 13 mL elution volume in **(B)**. Black scale bar at the bottom right represents 100 nm.

A

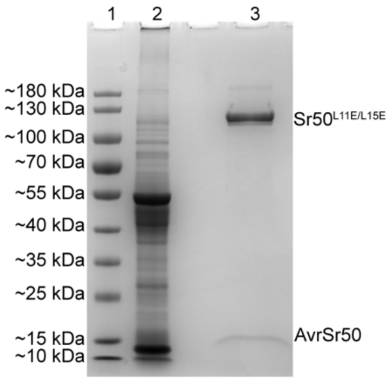

B

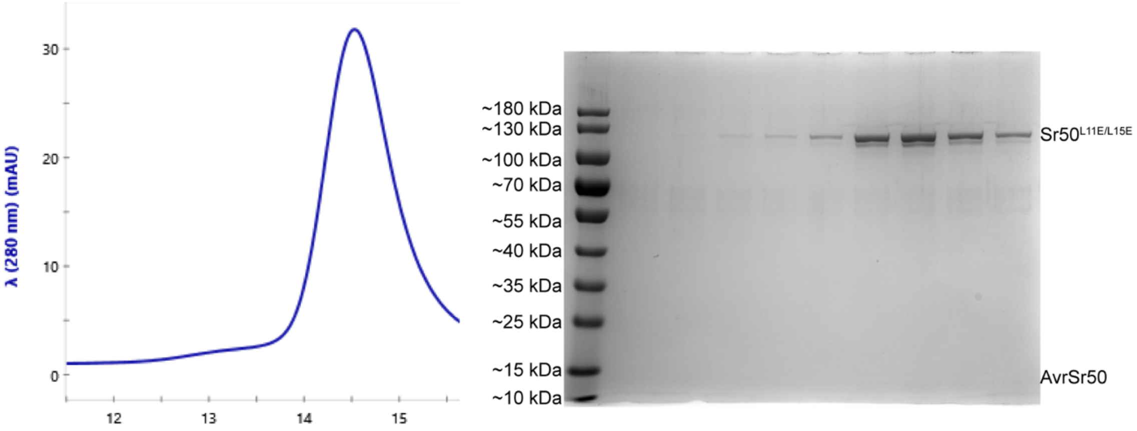

C

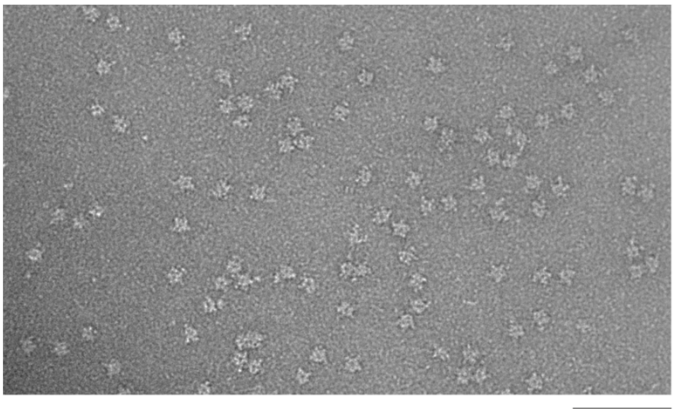

Appendix Figure S4. Transient expression and purification of the Sr50 resistosome from leaves of *N. benthamiana*.

(A) CBB-stained, SDS PAGE gel of a single step affinity purification of the $Sr50^{L11E/L15E}$ resistosome *via* the C-terminal, Twin-Strep-tag on Sr50. Lane #1: ladder; lane #2: lysate (5 µL loaded); lane #3: first-step eluate (45 µL/1 mL loaded). Samples run on a 10% gel. **(B)** SEC profile (left) and accompanying SDS PAGE of resulting elution fractions (right). Samples run on a 10% gel. **(C)** Negative staining of a five-fold dilution from the fraction corresponding to the ~14.5 mL elution volume in **(B)**. Black scale bar at the bottom right represents 100 nm.

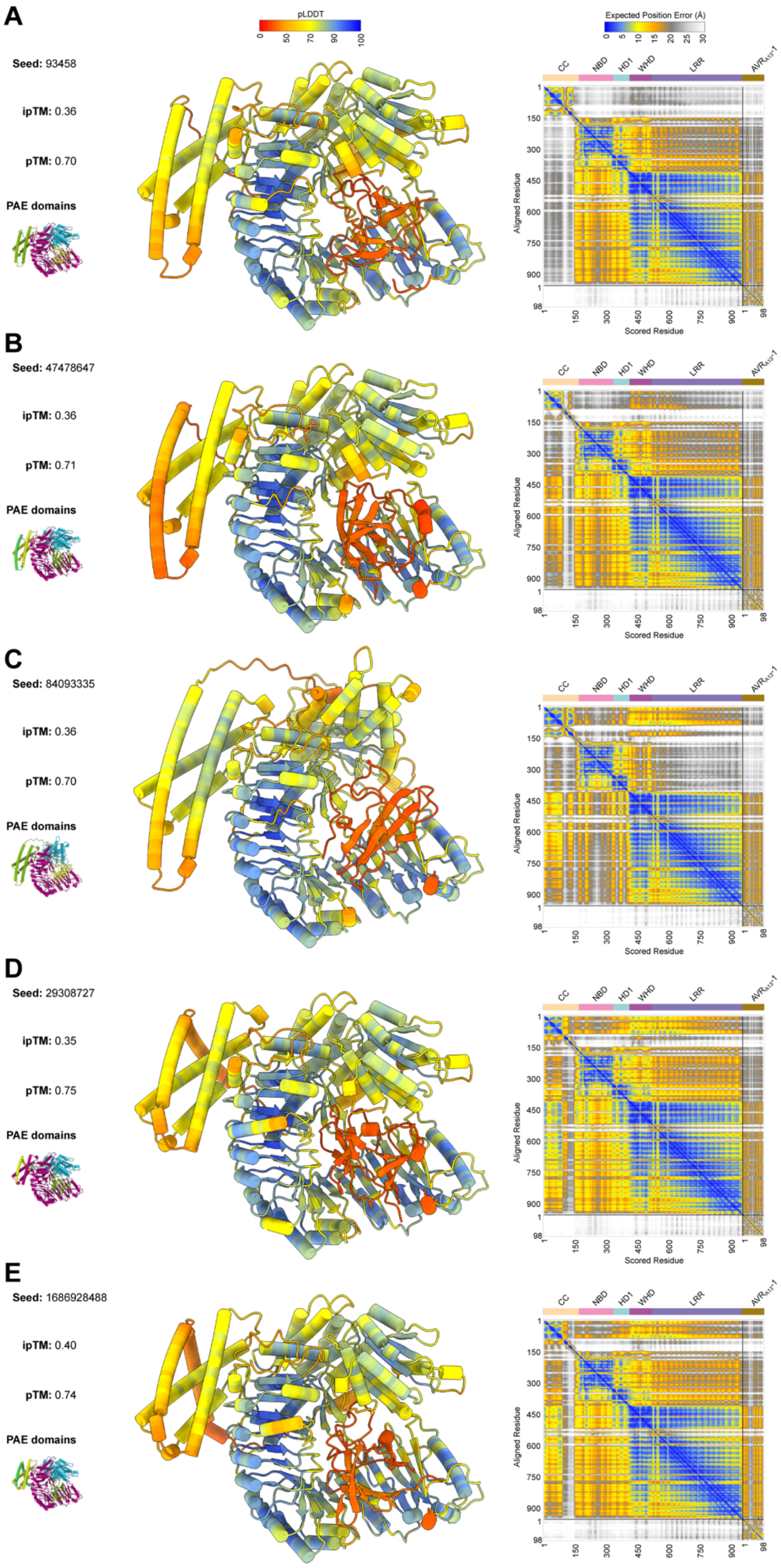

Appendix Figure S5. Overview of the AlphaFold 3 quality scores of the five predicted models of the MLA13-AVR$_{A13}$-1 complex presented in main Figure 2.

Note that while the predicted aligned error (PAE) highlights the uncertainty about the relative position of AVR$_{A13}$-1 in the complex, the uncertainty of the NBD position is not that obvious in most of the PAE plots. Also note that AVR$_{A13}$-1 is generally modelled with very low confidence, as observable by the predicted local distance difference test (pLDDT) scores of lower than 50.

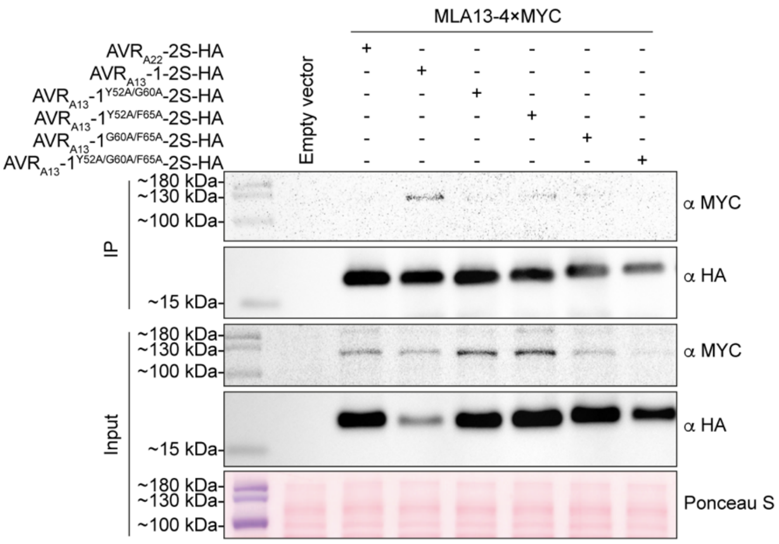

Appendix Figure S6. Co-IP assays of AVR$_{A13}$-1 interface substitution mutants that result in a reduced or loss of cell death activity as presented in main Figure 3.

Protein was immunoprecipitated *via* the Twin-Strep-tag on AVR$_{A13}$-1. Samples were run on a 12% SDS PAGE gel.

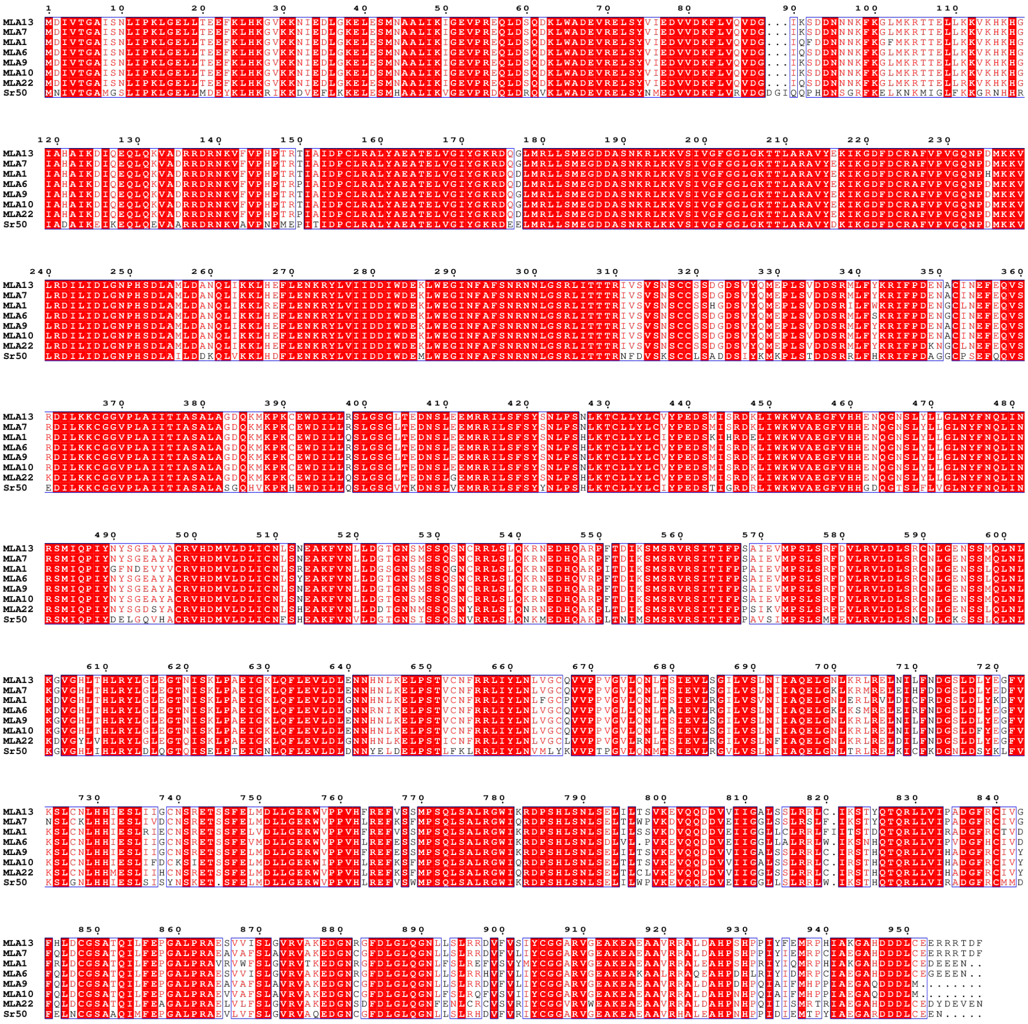

Appendix Figure S7. Amino acid sequence alignment of all the MLAs that recognise a matching *Bh* effector and Sr50.

Alignment performed using MUSCLE and visualised using ESPript 3.0.

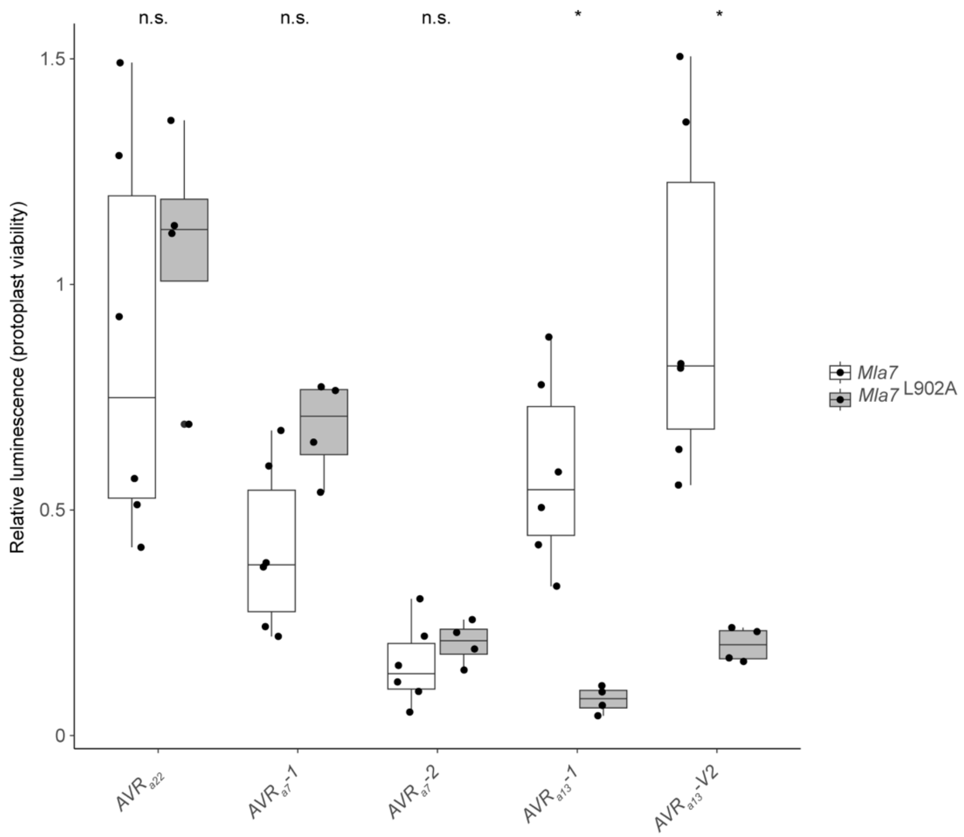

Appendix Figure S8. The MLA7^{L902A} substitution mutant results in expanded effector recognition, similar to MLA7^{L902S} as presented in main Figure 5.

Co-expression of MLA7 (data from main Figure 5) and MLA7^{L902A} with effector variants in barley protoplasts. Luminescence is normalised to EV + MLA7 (= 1) or EV + MLA7^{L902S} (= 1). High relative luminescence suggests low cell death response and therefore suggests low effector interaction with the receptor. The six data points represent two technical replicates performed with three independently prepared protoplast samples. Treatments labelled with an asterisk differ significantly (p <0.05) according to the Welch two-sample t-test. The p values for AVR_{a22}, AVR_{a7}-1, AVR_{a7}-2, AVR_{a13}-1 and AVR_{a13}-V2 are 0.393, 0.022, 0.314, 0.002 and 0.005, respectively. In the box plot, the top, middle, and bottom horizontal lines of the box correspond to the upper quartile, the median, and the lower quartile, respectively. The whiskers extend to the smallest and largest data points within 1.5 times the interquartile range from Q1 and Q3. Any points outside this range are plotted as dots and considered outliers.

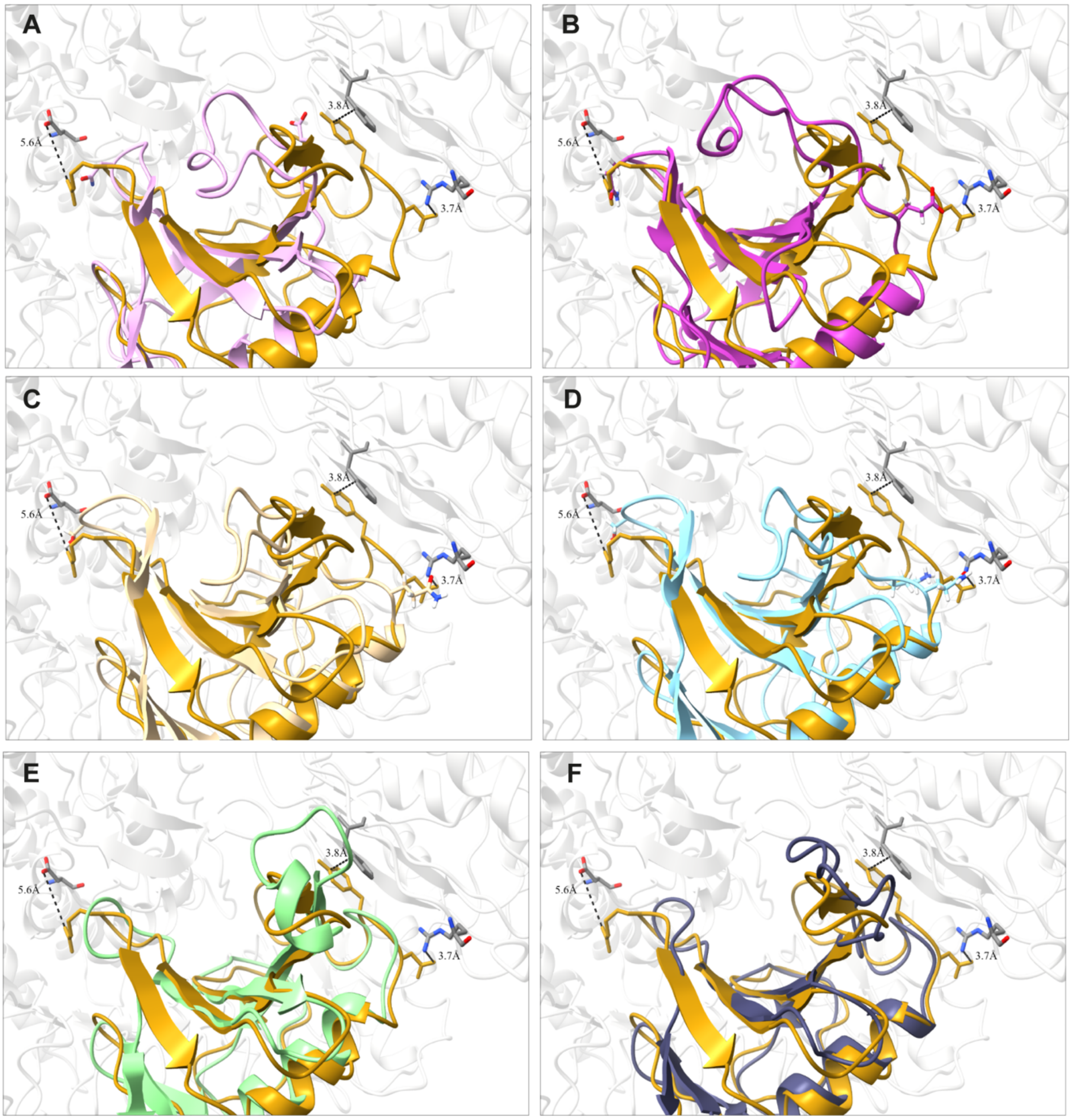

Appendix Figure S9. Structural alignment of $\mathrm{AVR}_{\mathrm{A13}}$-1 in the heterodimer with the crystal structures of other effectors.

Experimentally tested residues on $\mathrm{AVR}_{\mathrm{A13}}$-1 (dark goldenrod colour) that are believed to interact with MLA13 (transparent grey) are highlighted with proximity labels. Aligned effectors include: **(A)** $\mathrm{AVR}_{\mathrm{A6}}$, **(B)** $\mathrm{AVR}_{\mathrm{A7}}$-2, **(C)** $\mathrm{AVR}_{\mathrm{A10}}$, **(D)** $\mathrm{AVR}_{\mathrm{A22}}$, **(E)** CSEP0064, **(F)** AvrPm2.

Appendix Table S1. Cryo-EM parameters and statistical output for the MLA13-AVR$_{A13}$ heterodimer.

**Sample conditions**		
Grid type	Quantifoil Cu R2/4 (200 mesh) + Graphene Oxide	
**Cryo-EM data collection**		
Microscope	Titan Krios G3i	
Voltage (kV)	300	
Spherical aberration (mm)	2.7	
Condenser C2 aperture (μm)	70	
Objective aperture size (μm)	100	
Camera	Falcon III	
Pixel size	0.862	
Total dose (electron*$Å^{-2}$)	42	
Number of frames	42	
Images per hole	3	
Energy filter	None	
Defocus range (μm)	-2.0 to -0.3	
# micrographs collected	8,188	
# micrographs used	5,897	
**Cryo-EM data processing**		
software	cryosparc v4.4.1+patch240110	
Particles<br>after 2D classification<br>after 3D sorting	 115,686<br>48,191	
Resolution (FSC 0.143, Å)	3.8	
**Model building and refinement**		
Software for building	Coot 0.9.4.7 EL	
Residues build<br>MLA13<br>AVRa13	 2-131,143-541,555-956<br>25-122	
Software for refinement	PHENIX 1.21 - 5207	
Composition (#)<br>Chains<br>Atoms<br>Residues<br>Water<br>Ligands	 2<br>8127 (Hydrogens: 0)<br>Protein: 1029<br>0<br>0	
Bonds (RMSD)<br>Length (Å) (# > 4σ)<br>Angles (°) (# > 4σ)	 0.002 (0)<br>0.518 (1)	
MolProbity score	2.04	
Clash score	8.96	
Ramachandran plot (%)<br>Outliers<br>Allowed<br>Favored	 0.10<br>10.19<br>89.72	
Rama-Z (Z-score, RMSD)<br>whole (N = 1021)<br>helix (N = 331)<br>sheet (N = 156)<br>loop (N = 534)	 -2.06 (0.26)<br>-0.17 (0.29)<br>-0.63 (0.46)<br>-2.39 (0.25)	
Rotamer Outliers (%)	0.0	
Peptide plane (%)<br>Cis proline/general<br>Twisted proline/general	 2.8/0.0<br>0.0/0.0	
CaBLAM outliers (%)	6.81	
Supplied Resolution (Å)	3.5	
Resolution Estimates (Å)<br>d 99 (full)<br>d model<br>d FSC model (0/0.143/0.5)	Masked<br>2.5<br>2.2<br>1.7/2.0/3.7	Unmasked<br>2.5<br>2.2<br>1.7/2.0/3.7
Model vs. Data<br>CC (mask<br>CC (box)<br>CC (peaks)<br>CC (volume)	 0.71<br>0.71<br>0.69<br>0.72	
