# Supplementary figures and images for: The barley MLA13-AVRA13 heterodimer reveals principles for immunoreceptor recognition of RNase-like powdery mildew effectors
Source: EMBO J. 2025 Feb 13;44(11):3210–30. doi: 10.1038/s44318-025-00373-9 (PMC12130304; doi:10.1038/s44318-025-00373-9)

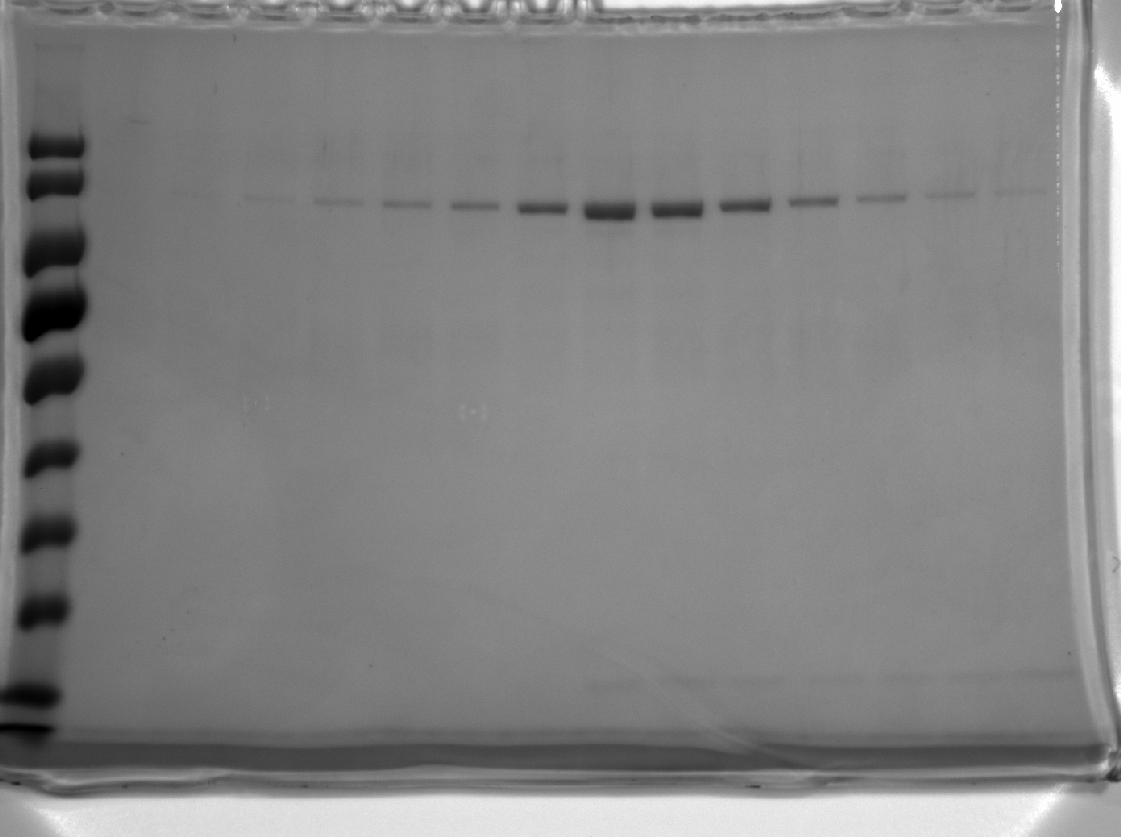

Supplement: Supplementary file 3 — Source data Fig. 1 [file 44318_2025_373_MOESM3_ESM.zip › EMBOJ-2024-118663_SD_Figure1/SD_Figure1A_2.tif]

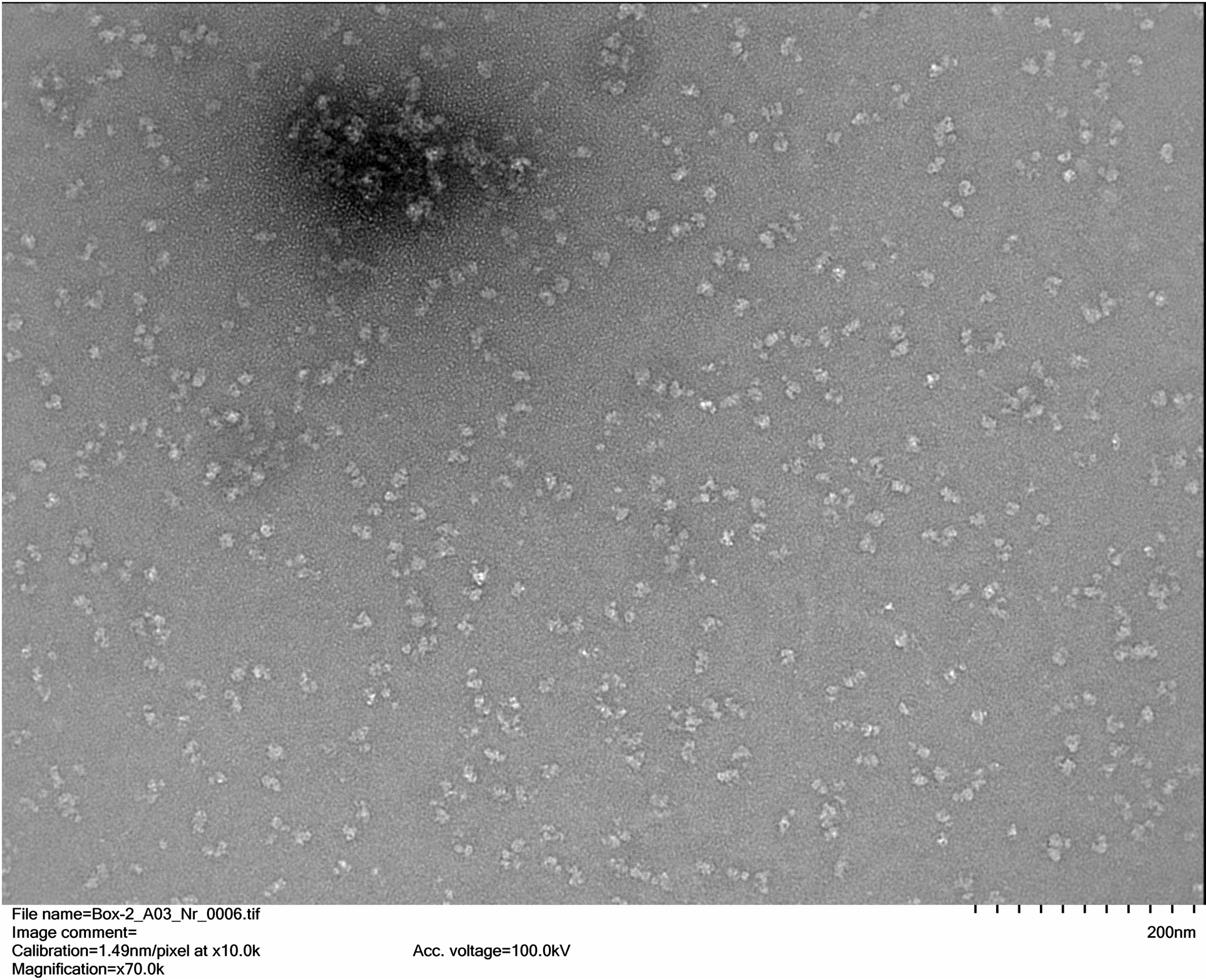

Supplement: Supplementary file 3 — Source data Fig. 1 [file 44318_2025_373_MOESM3_ESM.zip › EMBOJ-2024-118663_SD_Figure1/SD_Figure1B.png]

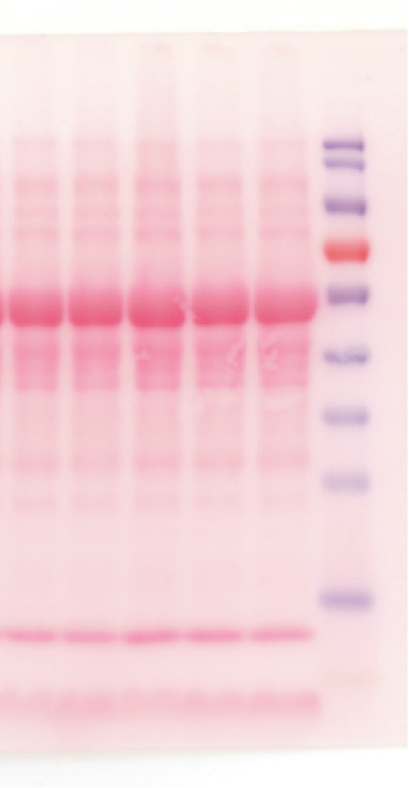

Supplement: Supplementary file 4 — Source data Fig. 3 [file 44318_2025_373_MOESM4_ESM.zip › EMBOJ-2024-118663_SD_Figure3/SD_Figure3D_4.tif]

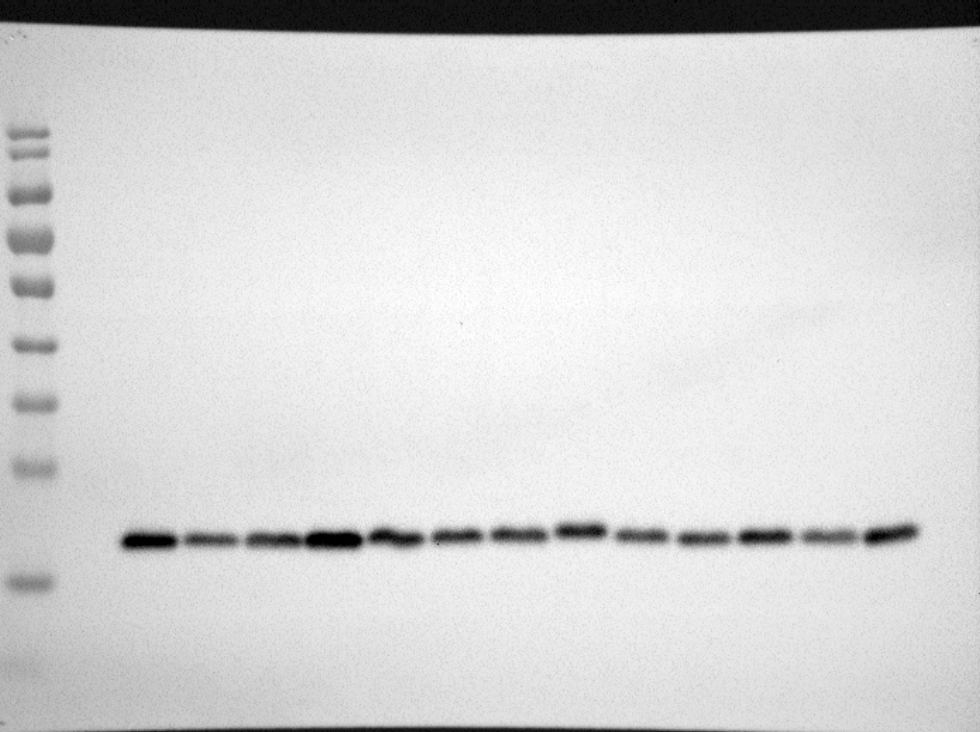

Supplement: Supplementary file 4 — Source data Fig. 3 [file 44318_2025_373_MOESM4_ESM.zip › EMBOJ-2024-118663_SD_Figure3/SD_Figure3D_1.tif]

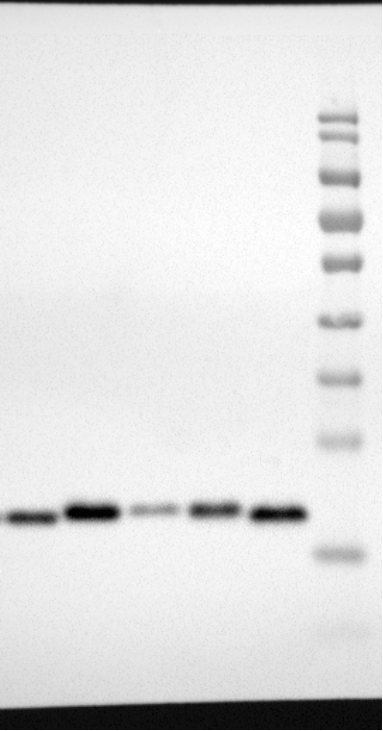

Supplement: Supplementary file 4 — Source data Fig. 3 [file 44318_2025_373_MOESM4_ESM.zip › EMBOJ-2024-118663_SD_Figure3/SD_Figure3D_2.tif]

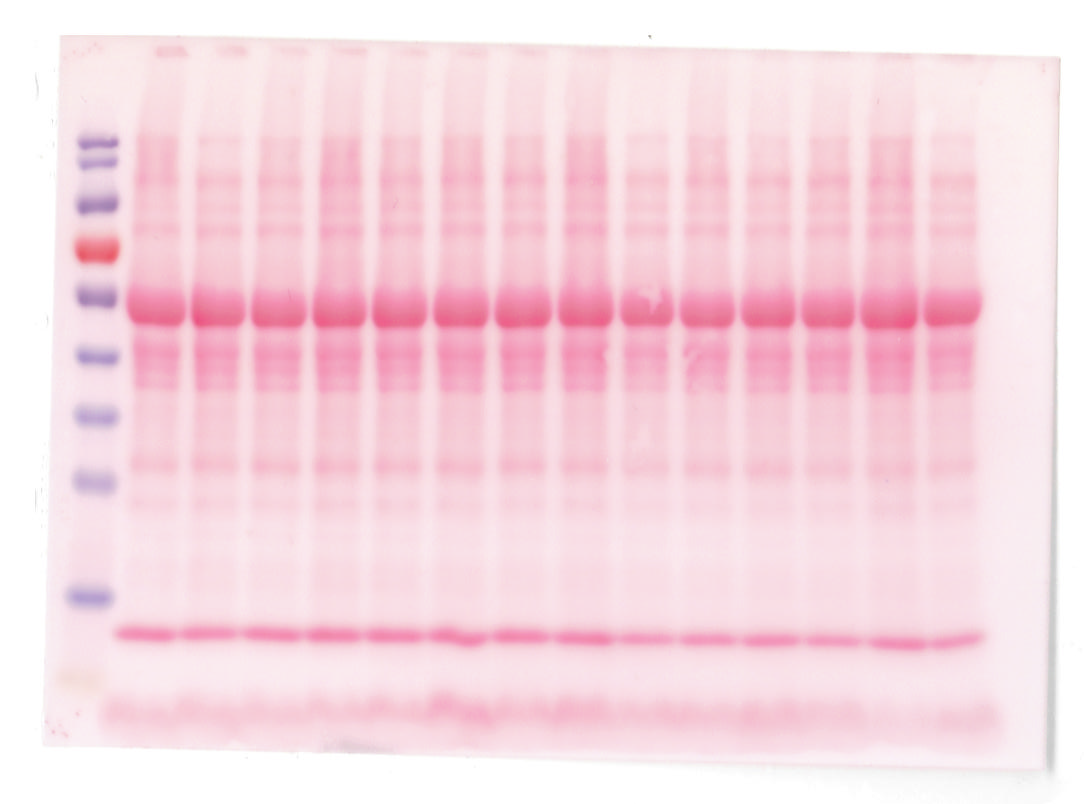

Supplement: Supplementary file 4 — Source data Fig. 3 [file 44318_2025_373_MOESM4_ESM.zip › EMBOJ-2024-118663_SD_Figure3/SD_Figure3D_3.tif]

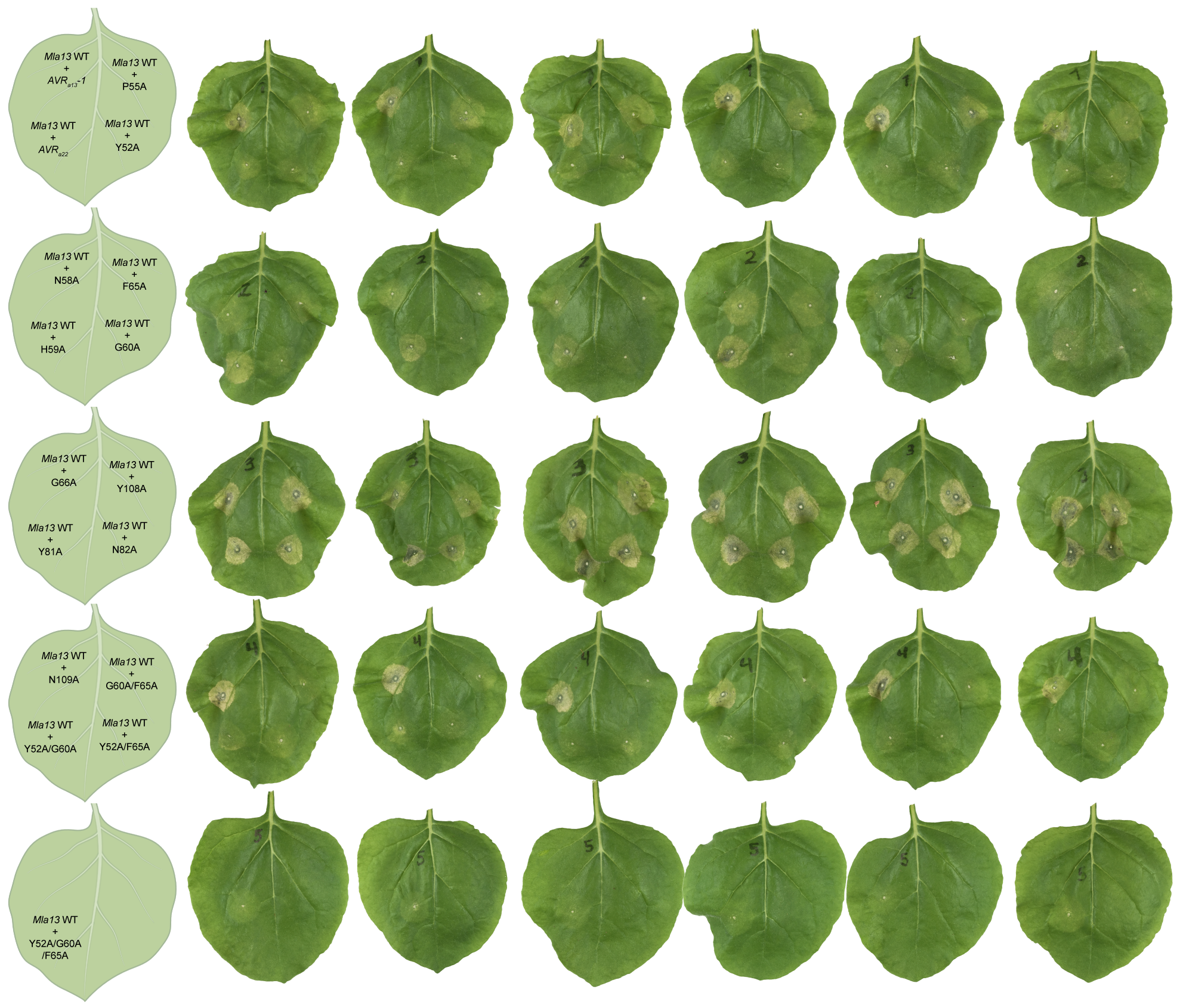

Supplement: Supplementary file 4 — Source data Fig. 3 [file 44318_2025_373_MOESM4_ESM.zip › EMBOJ-2024-118663_SD_Figure3/SD_Figure3C.tif]

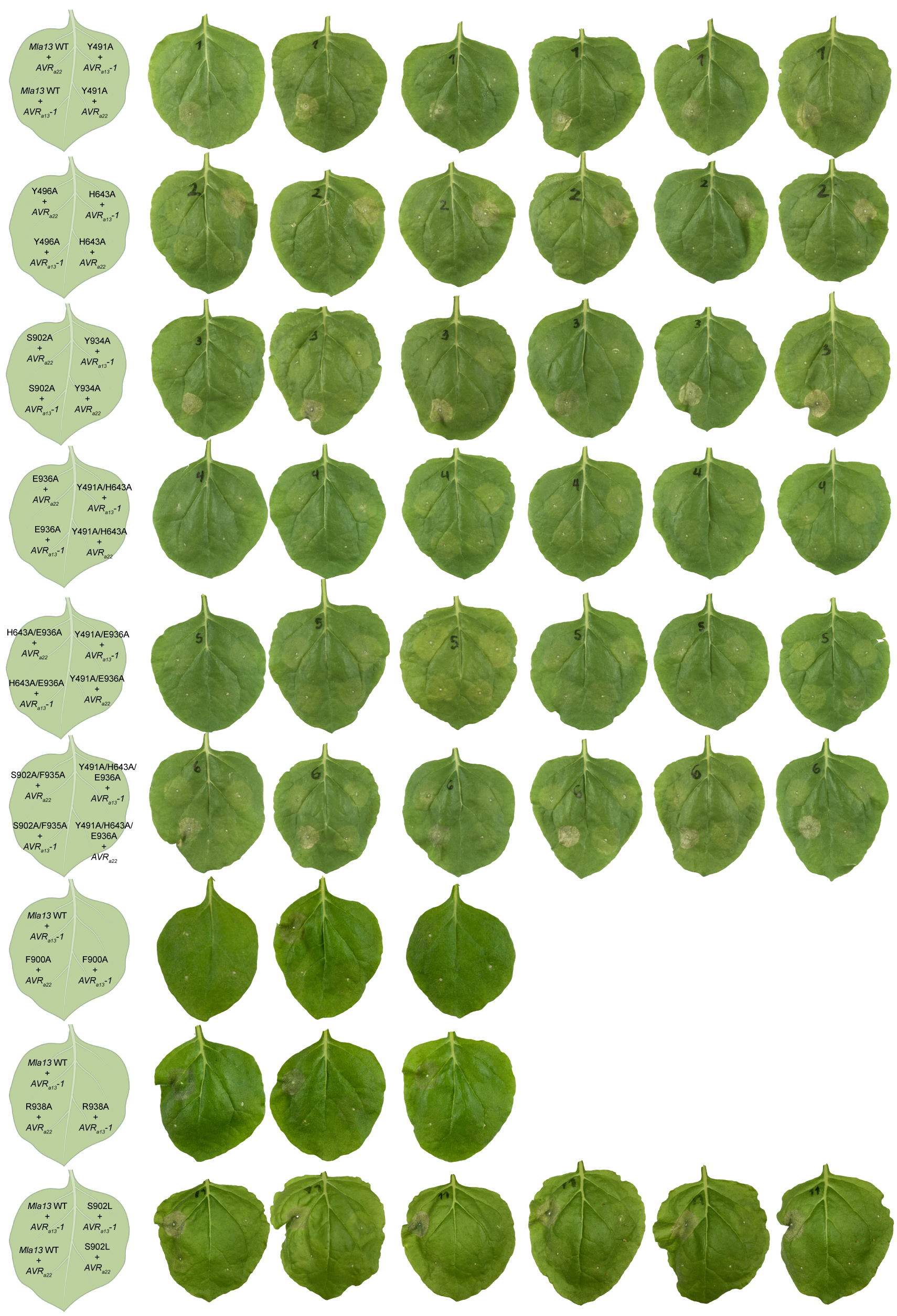

Supplement: Supplementary file 5 — Source data Fig. 4 [file 44318_2025_373_MOESM5_ESM.zip › EMBOJ-2024-118663_SD_Figure4/SD_Figure4C.tif]

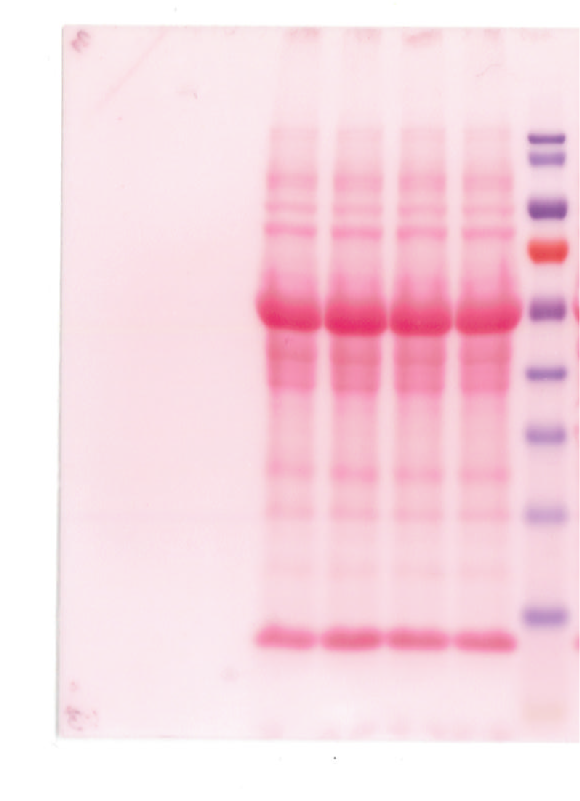

Supplement: Supplementary file 5 — Source data Fig. 4 [file 44318_2025_373_MOESM5_ESM.zip › EMBOJ-2024-118663_SD_Figure4/SD_Figure4D_4.tif]

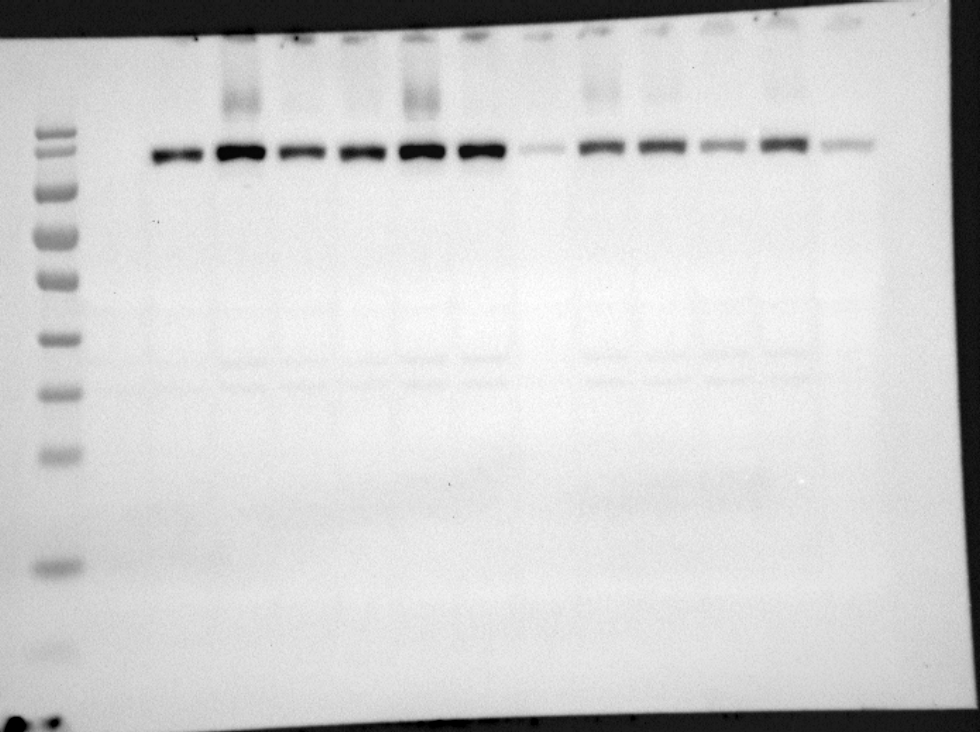

Supplement: Supplementary file 5 — Source data Fig. 4 [file 44318_2025_373_MOESM5_ESM.zip › EMBOJ-2024-118663_SD_Figure4/SD_Figure4D_1.tif]

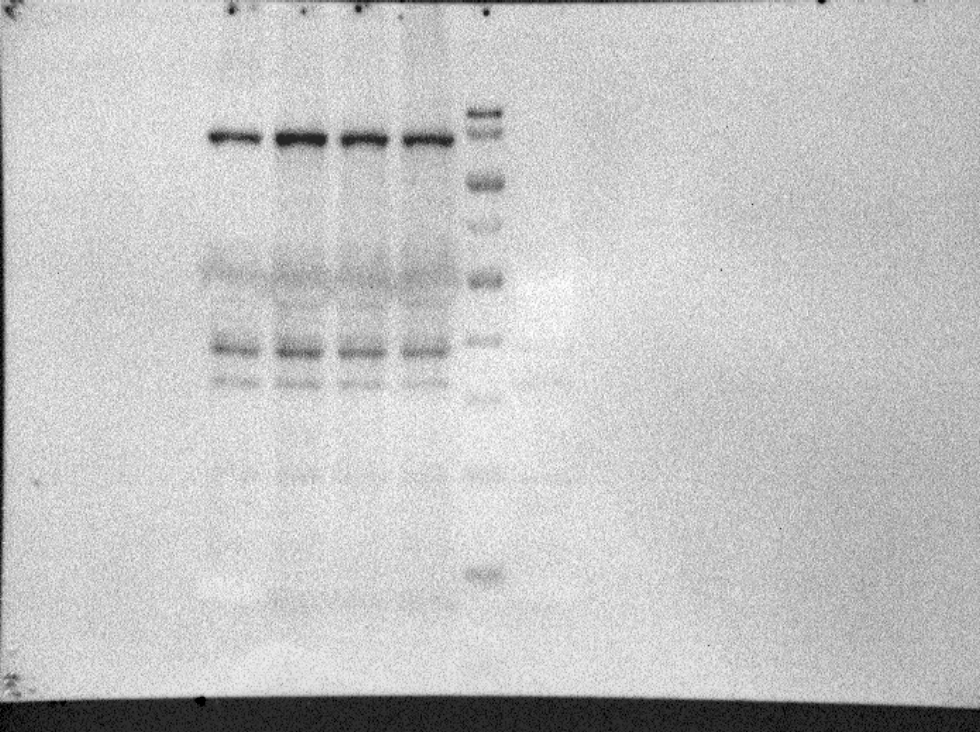

Supplement: Supplementary file 5 — Source data Fig. 4 [file 44318_2025_373_MOESM5_ESM.zip › EMBOJ-2024-118663_SD_Figure4/SD_Figure 4D_2.tif]

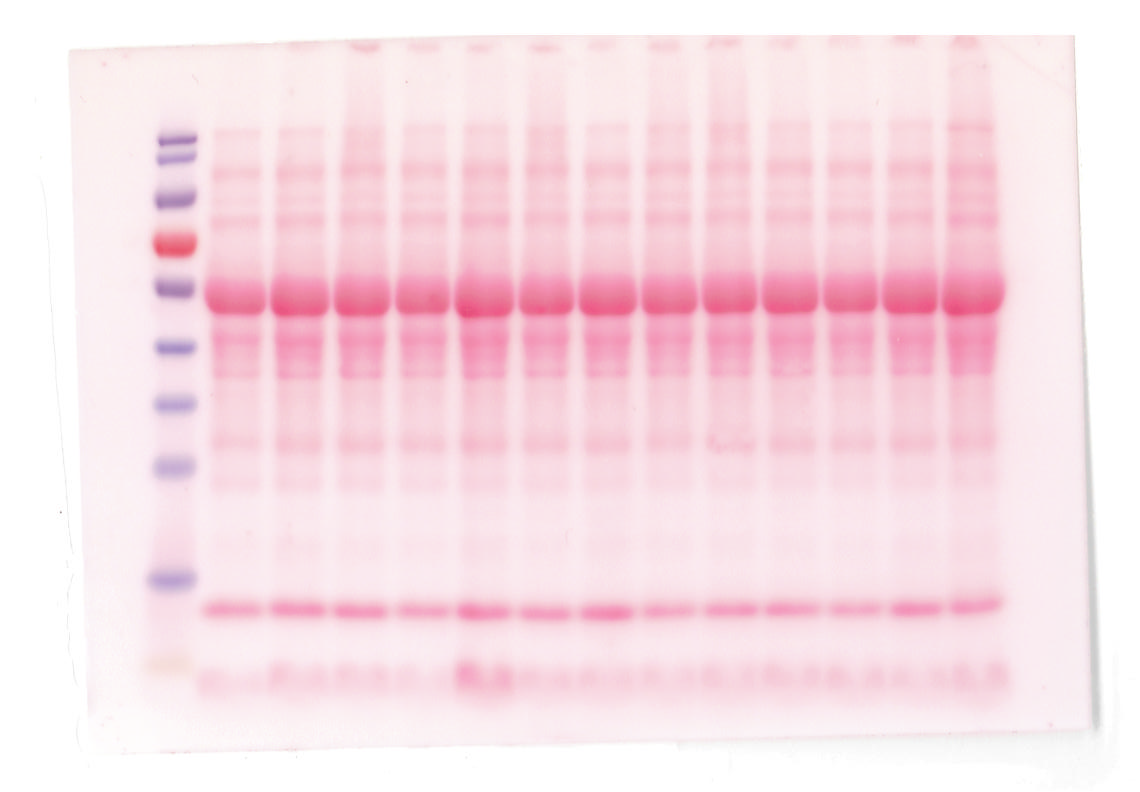

Supplement: Supplementary file 5 — Source data Fig. 4 [file 44318_2025_373_MOESM5_ESM.zip › EMBOJ-2024-118663_SD_Figure4/SD_Figure4D_3.tif]

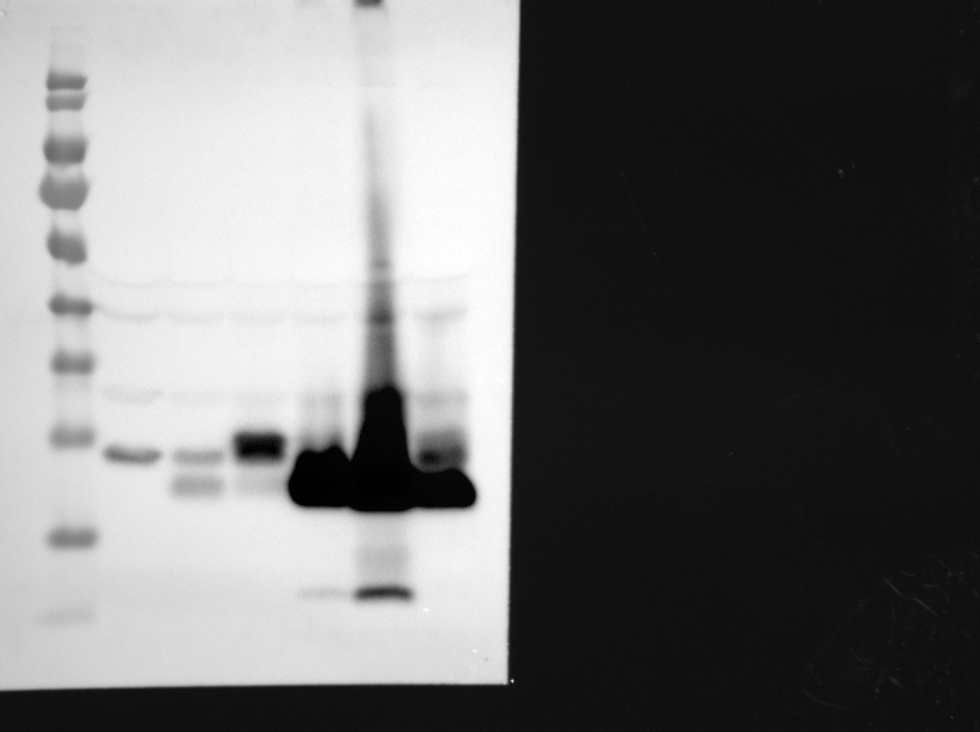

Supplement: Supplementary file 6 — Source data Fig. 5 [file 44318_2025_373_MOESM6_ESM.zip › EMBOJ-2024-118663_SD_Figure5/SD_Figure5C_1.tif]

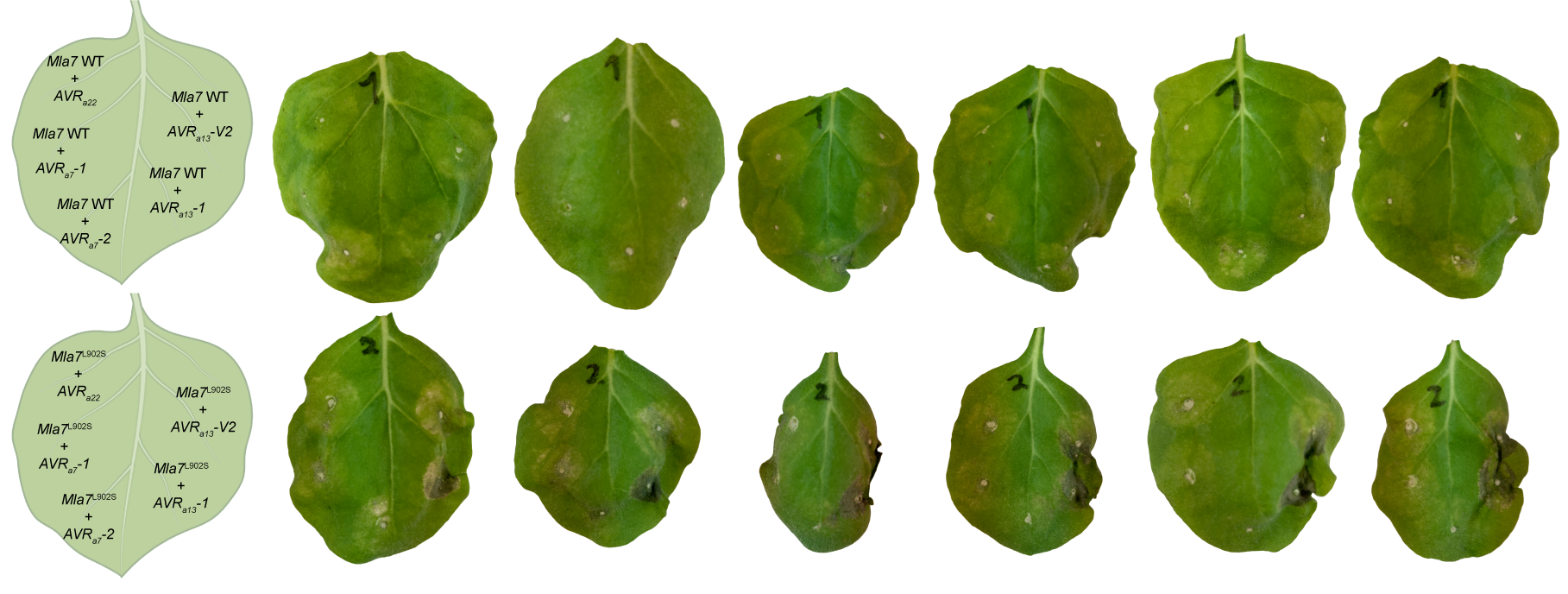

Supplement: Supplementary file 6 — Source data Fig. 5 [file 44318_2025_373_MOESM6_ESM.zip › EMBOJ-2024-118663_SD_Figure5/SD_Figure5B.tif]

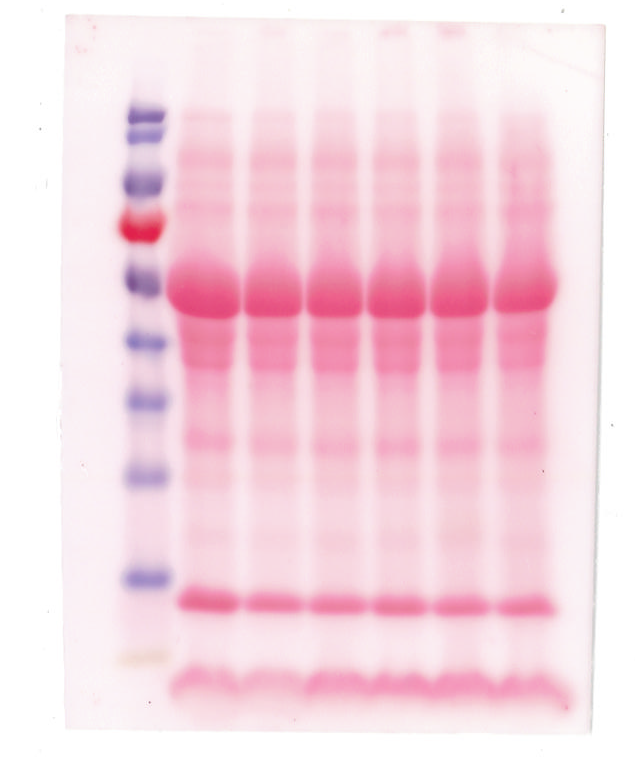

Supplement: Supplementary file 6 — Source data Fig. 5 [file 44318_2025_373_MOESM6_ESM.zip › EMBOJ-2024-118663_SD_Figure5/SD_Figure5C_2.tif]

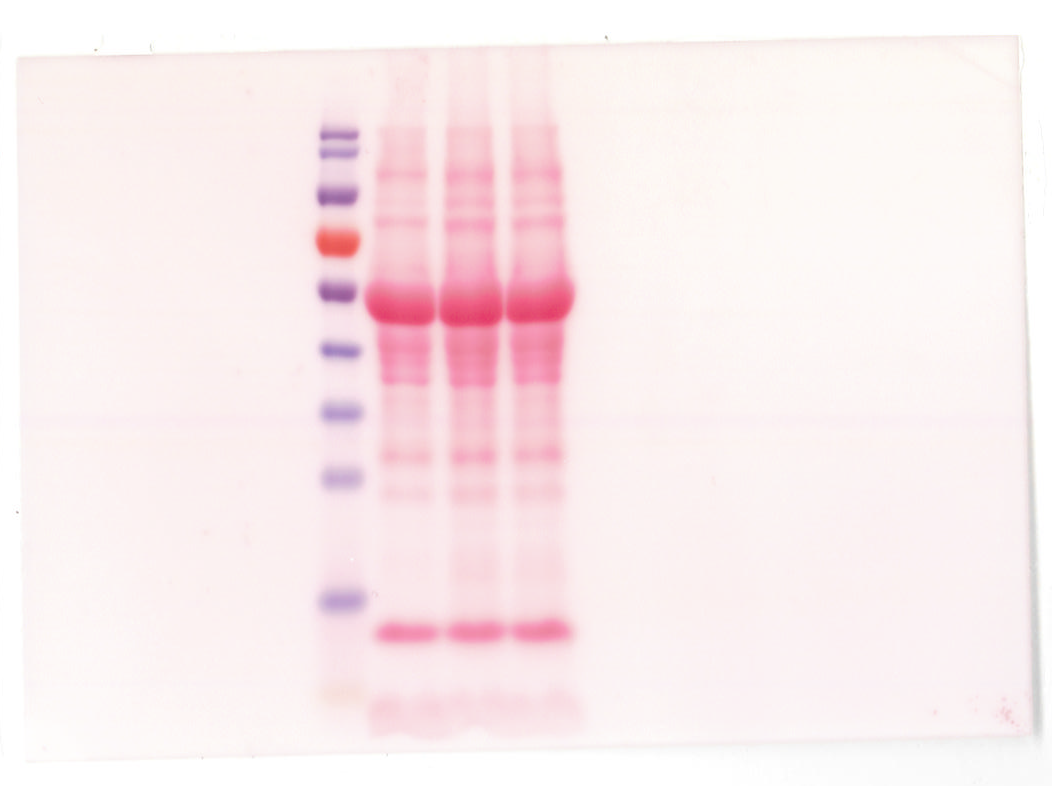

Supplement: Supplementary file 6 — Source data Fig. 5 [file 44318_2025_373_MOESM6_ESM.zip › EMBOJ-2024-118663_SD_Figure5/SD_Figure5D_2.tif]

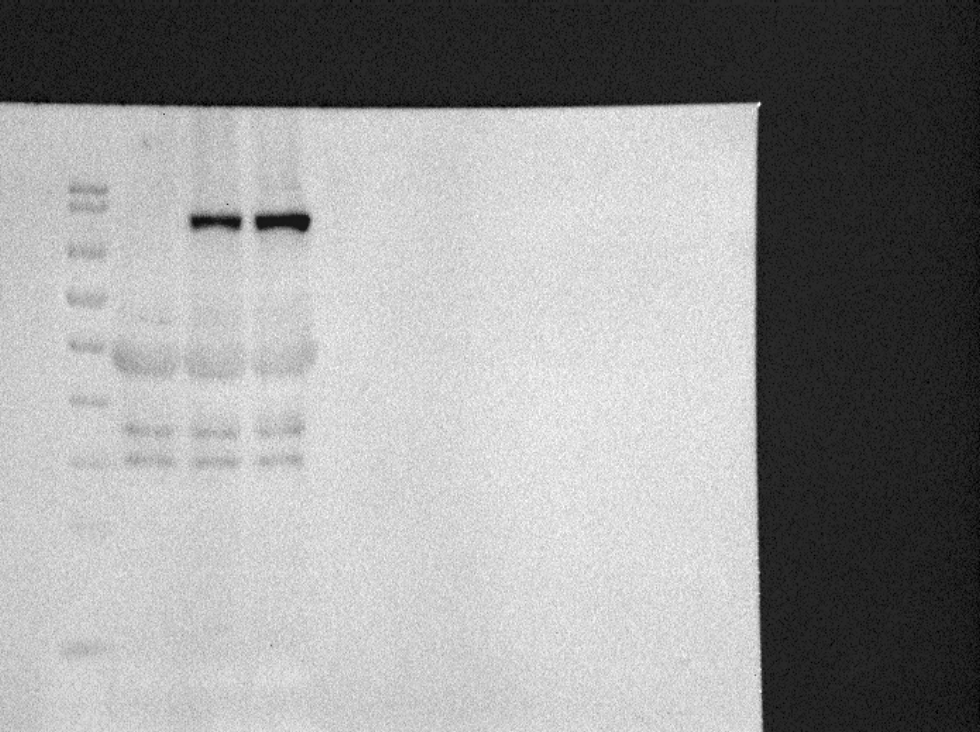

Supplement: Supplementary file 6 — Source data Fig. 5 [file 44318_2025_373_MOESM6_ESM.zip › EMBOJ-2024-118663_SD_Figure5/SD_Figure5D_1.tif]

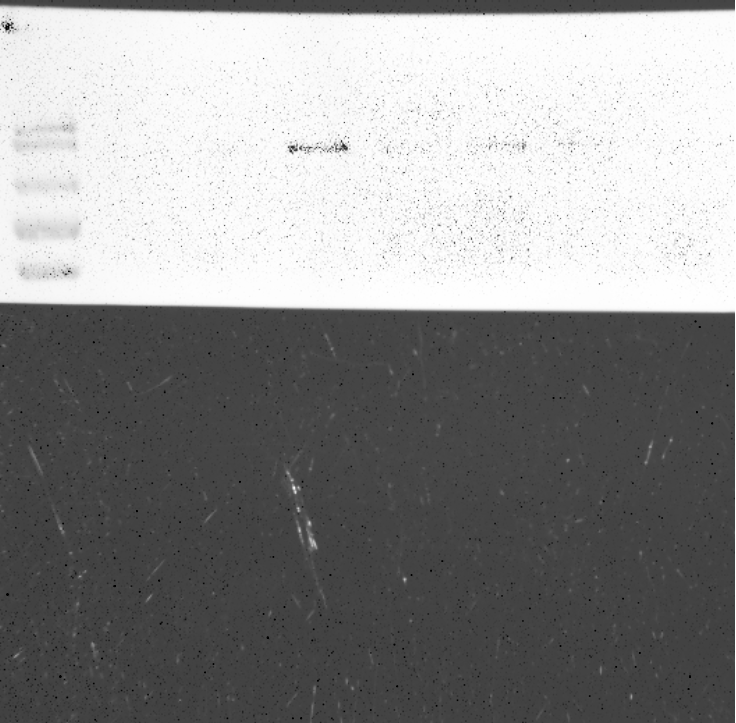

Supplement: Supplementary file 7 — EV/Appendix Source Data [file 44318_2025_373_MOESM7_ESM.zip › EMBOJ-2024-118663_SD_EV_AppendixFigures/SD_Appendix6_1.tif]

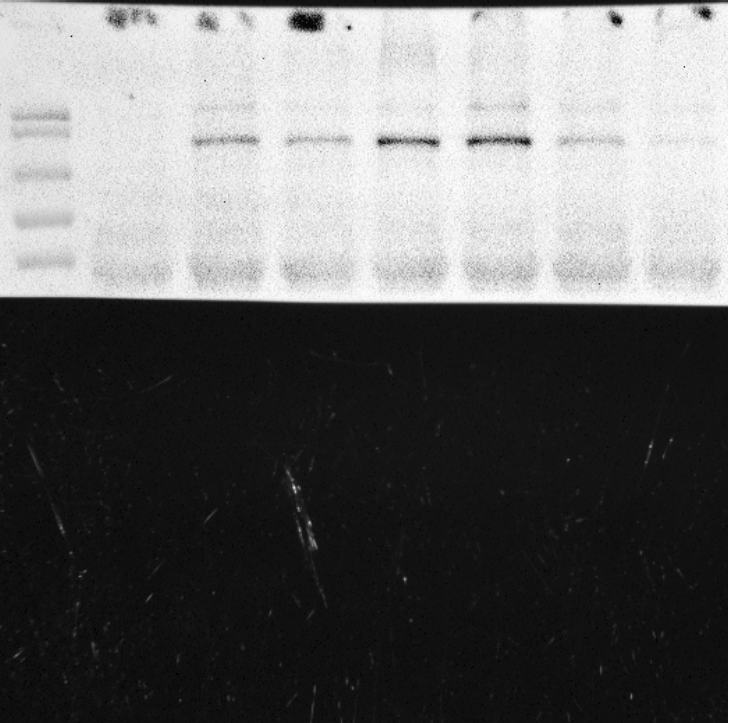

Supplement: Supplementary file 7 — EV/Appendix Source Data [file 44318_2025_373_MOESM7_ESM.zip › EMBOJ-2024-118663_SD_EV_AppendixFigures/SD_Appendix6_3.tif]

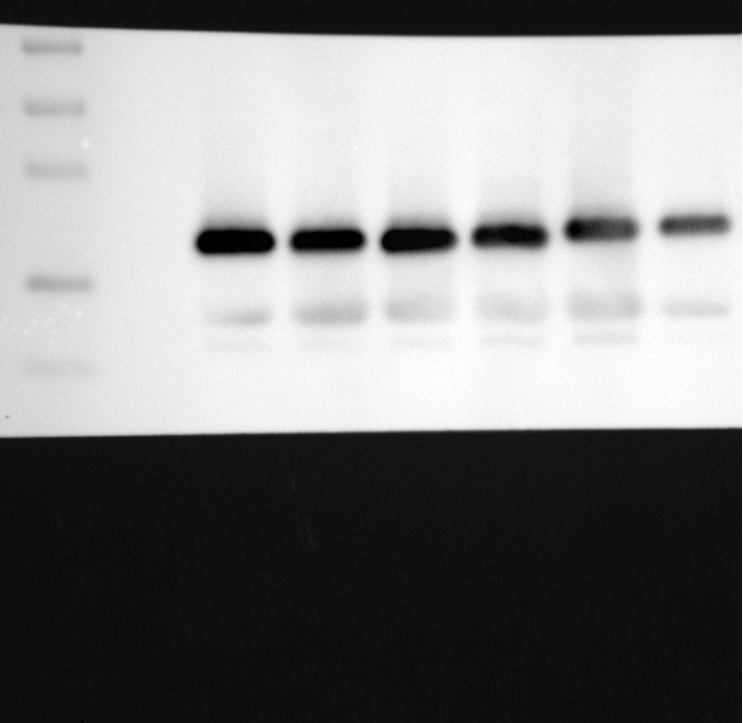

Supplement: Supplementary file 7 — EV/Appendix Source Data [file 44318_2025_373_MOESM7_ESM.zip › EMBOJ-2024-118663_SD_EV_AppendixFigures/SD_Appendix6_2.tif]

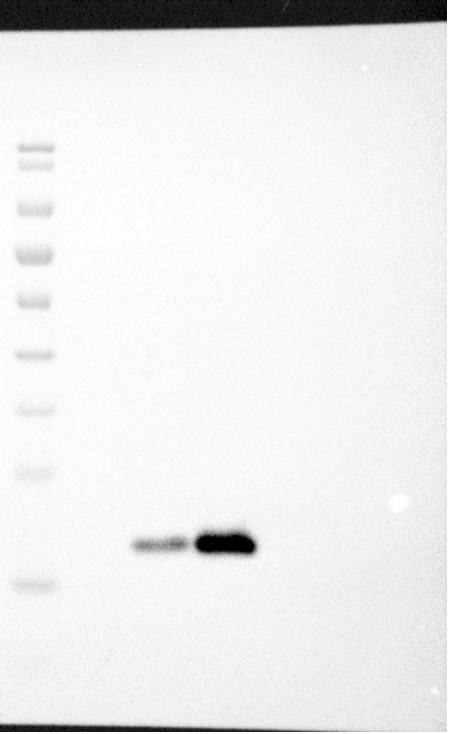

Supplement: Supplementary file 7 — EV/Appendix Source Data [file 44318_2025_373_MOESM7_ESM.zip › EMBOJ-2024-118663_SD_EV_AppendixFigures/SD_Appendix1C_1.tif]

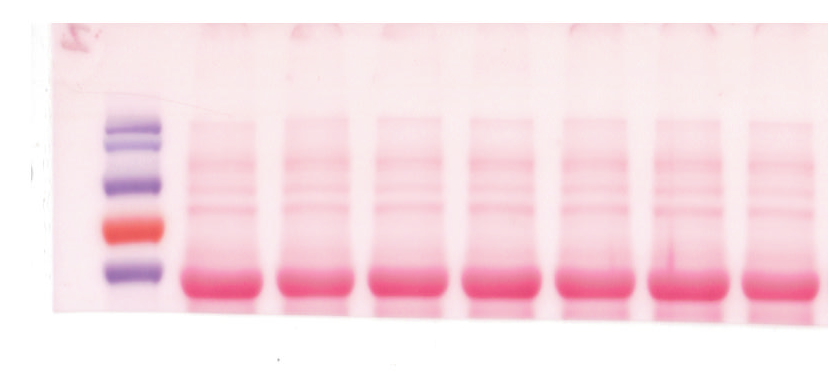

Supplement: Supplementary file 7 — EV/Appendix Source Data [file 44318_2025_373_MOESM7_ESM.zip › EMBOJ-2024-118663_SD_EV_AppendixFigures/SD_Appendix6_5.tif]

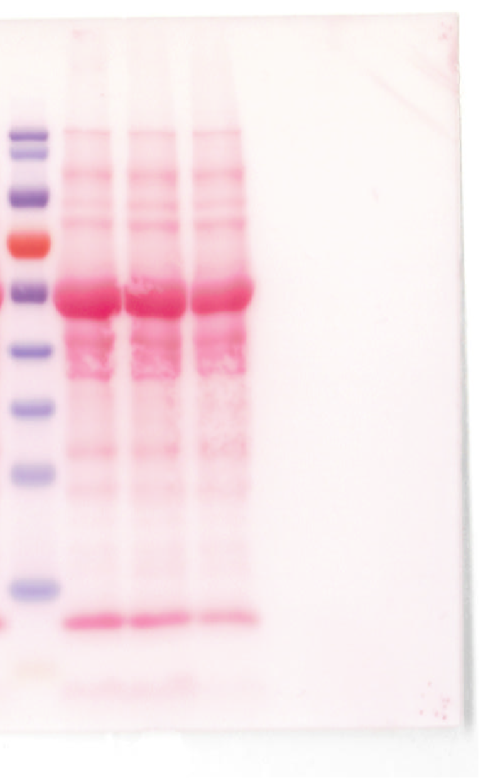

Supplement: Supplementary file 7 — EV/Appendix Source Data [file 44318_2025_373_MOESM7_ESM.zip › EMBOJ-2024-118663_SD_EV_AppendixFigures/SD_Appendix1C_2.tif]

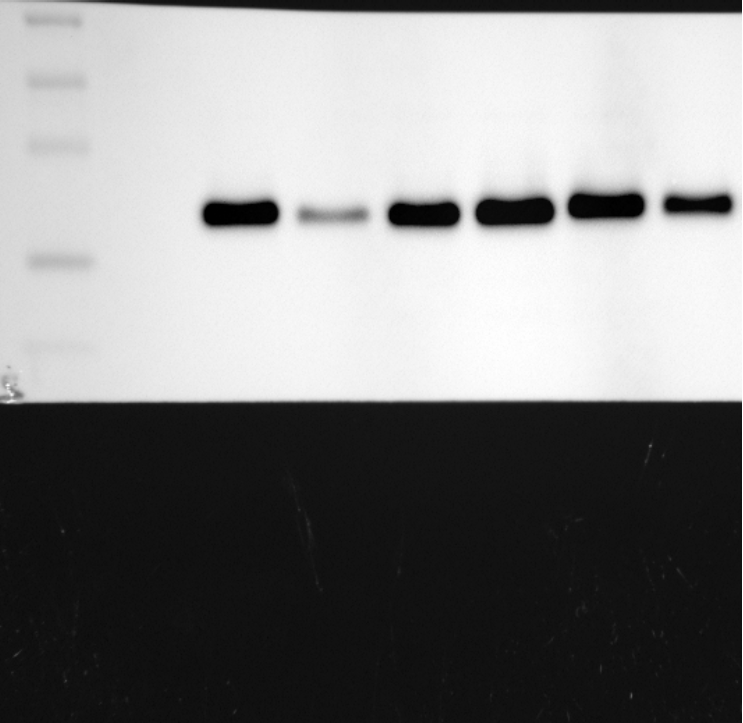

Supplement: Supplementary file 7 — EV/Appendix Source Data [file 44318_2025_373_MOESM7_ESM.zip › EMBOJ-2024-118663_SD_EV_AppendixFigures/SD_Appendix6_4.tif]

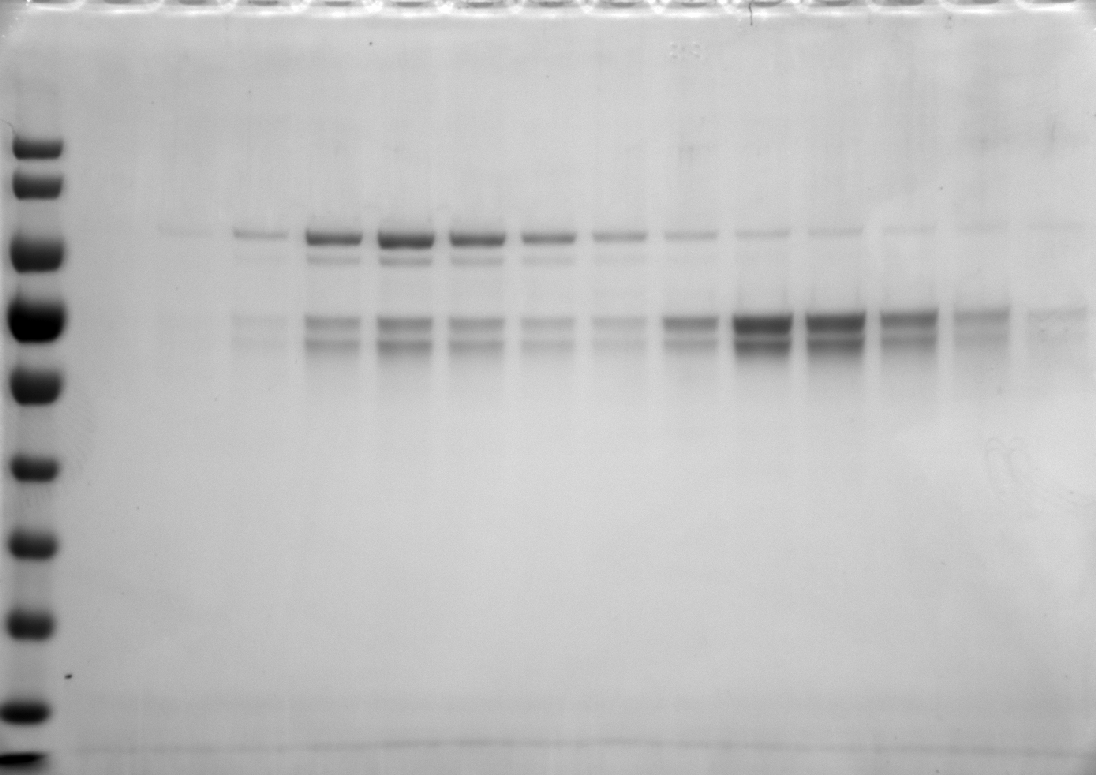

Supplement: Supplementary file 7 — EV/Appendix Source Data [file 44318_2025_373_MOESM7_ESM.zip › EMBOJ-2024-118663_SD_EV_AppendixFigures/SD_Appendix3B_2.tif]

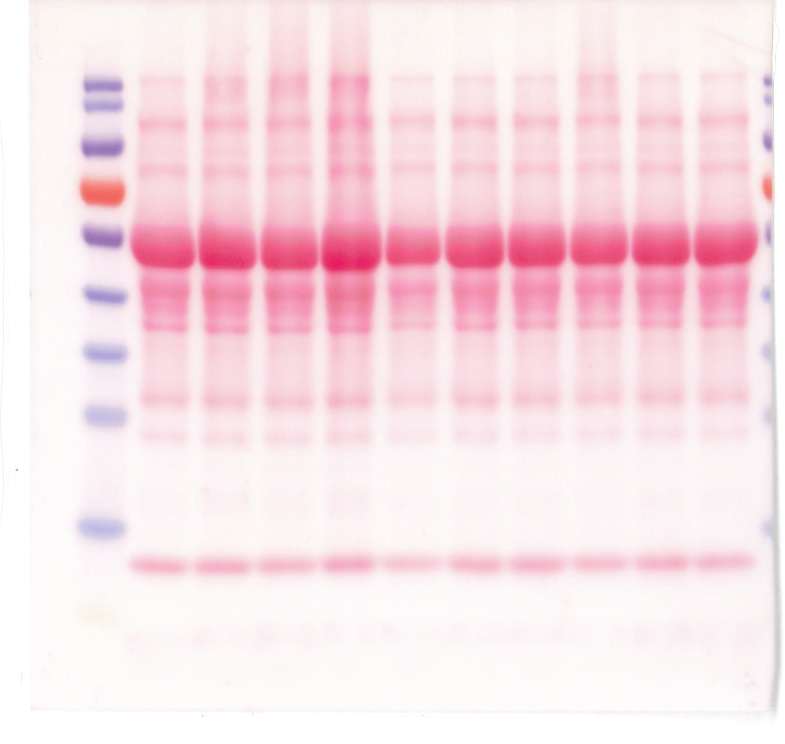

Supplement: Supplementary file 7 — EV/Appendix Source Data [file 44318_2025_373_MOESM7_ESM.zip › EMBOJ-2024-118663_SD_EV_AppendixFigures/SD_Appendix1B_2.tif]

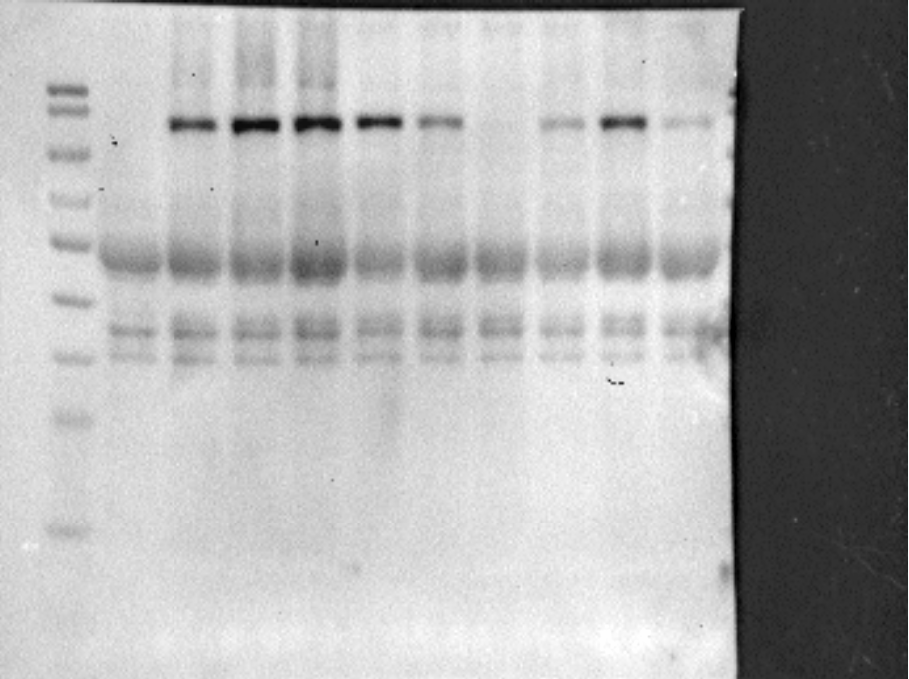

Supplement: Supplementary file 7 — EV/Appendix Source Data [file 44318_2025_373_MOESM7_ESM.zip › EMBOJ-2024-118663_SD_EV_AppendixFigures/SD_Appendix1B_1.tif]

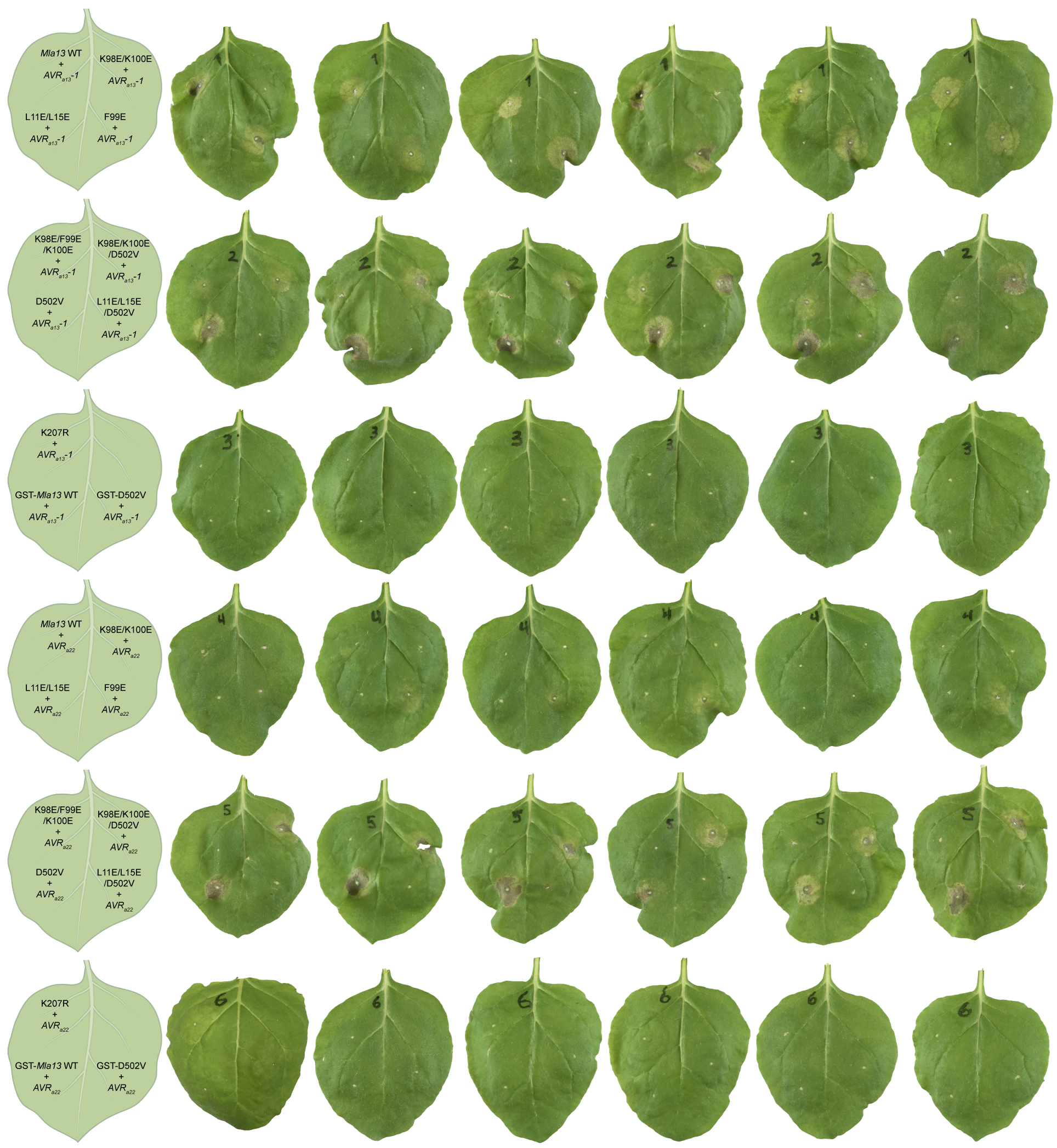

Supplement: Supplementary file 7 — EV/Appendix Source Data [file 44318_2025_373_MOESM7_ESM.zip › EMBOJ-2024-118663_SD_EV_AppendixFigures/SD_Appendix1A.tif]

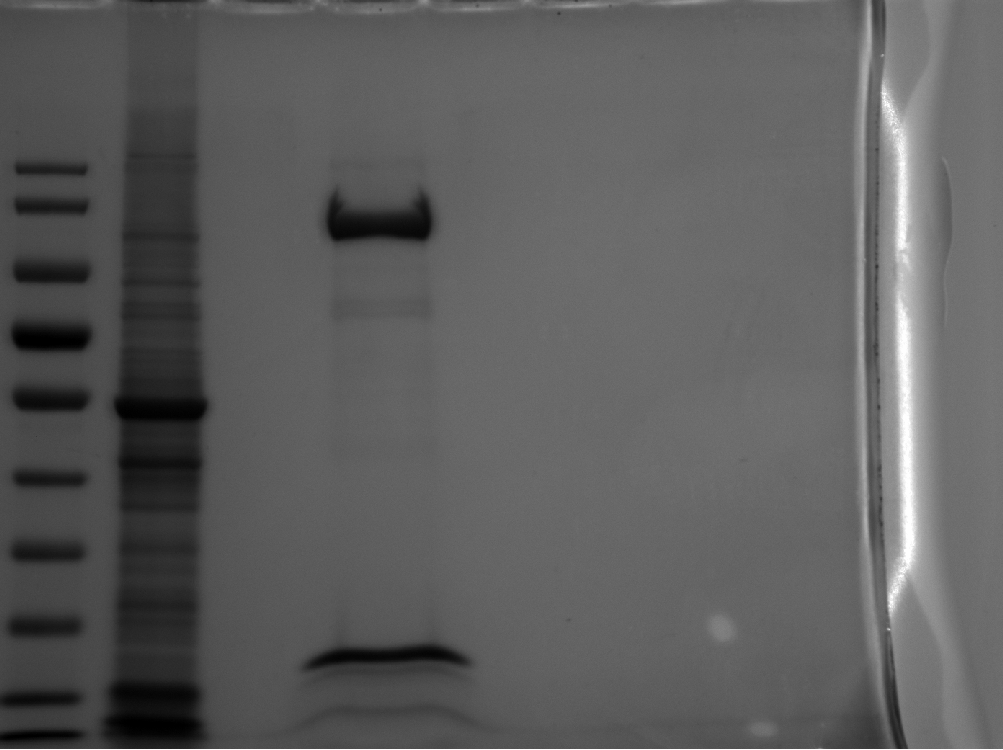

Supplement: Supplementary file 7 — EV/Appendix Source Data [file 44318_2025_373_MOESM7_ESM.zip › EMBOJ-2024-118663_SD_EV_AppendixFigures/SD_Appendix2A.tif]

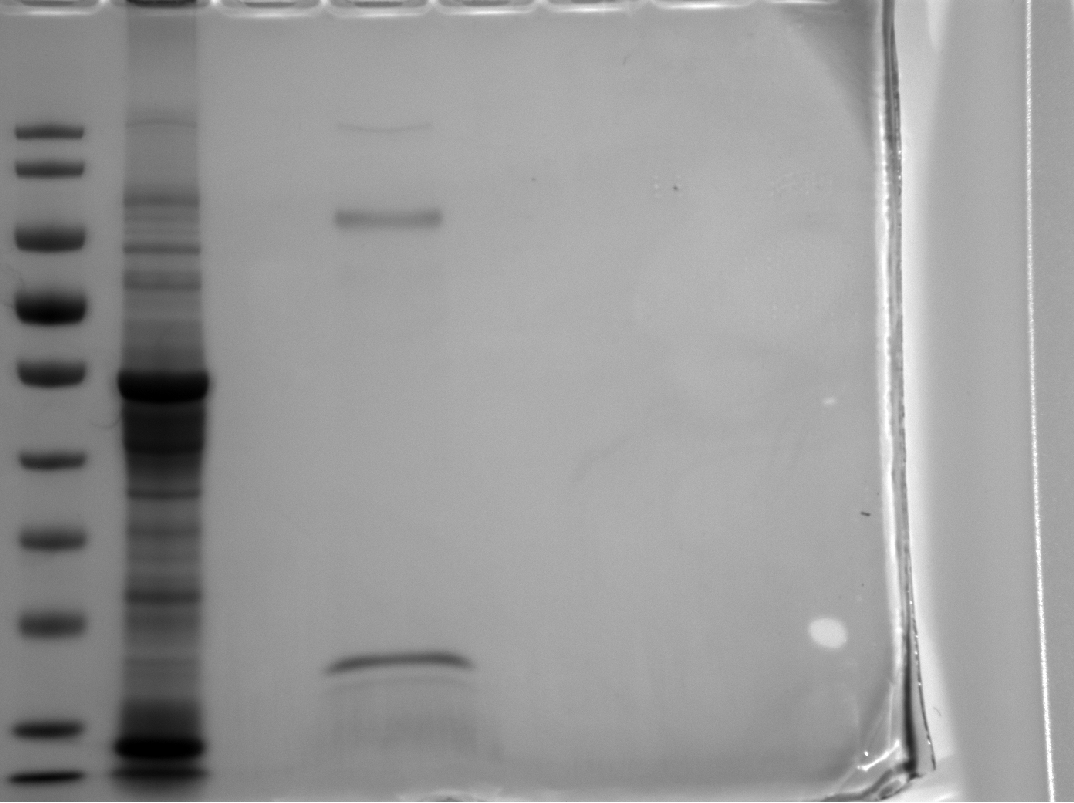

Supplement: Supplementary file 7 — EV/Appendix Source Data [file 44318_2025_373_MOESM7_ESM.zip › EMBOJ-2024-118663_SD_EV_AppendixFigures/SD_FigureEV1E_2.tif]

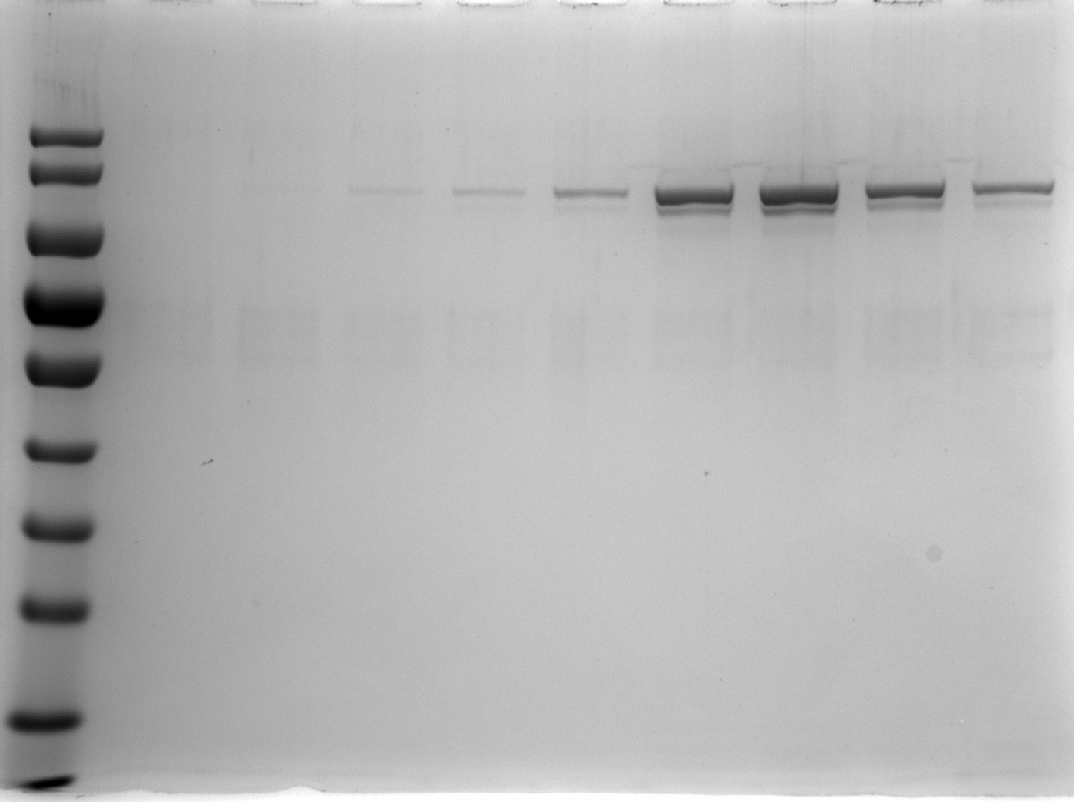

Supplement: Supplementary file 7 — EV/Appendix Source Data [file 44318_2025_373_MOESM7_ESM.zip › EMBOJ-2024-118663_SD_EV_AppendixFigures/SD_Appendix4B_2.tif]

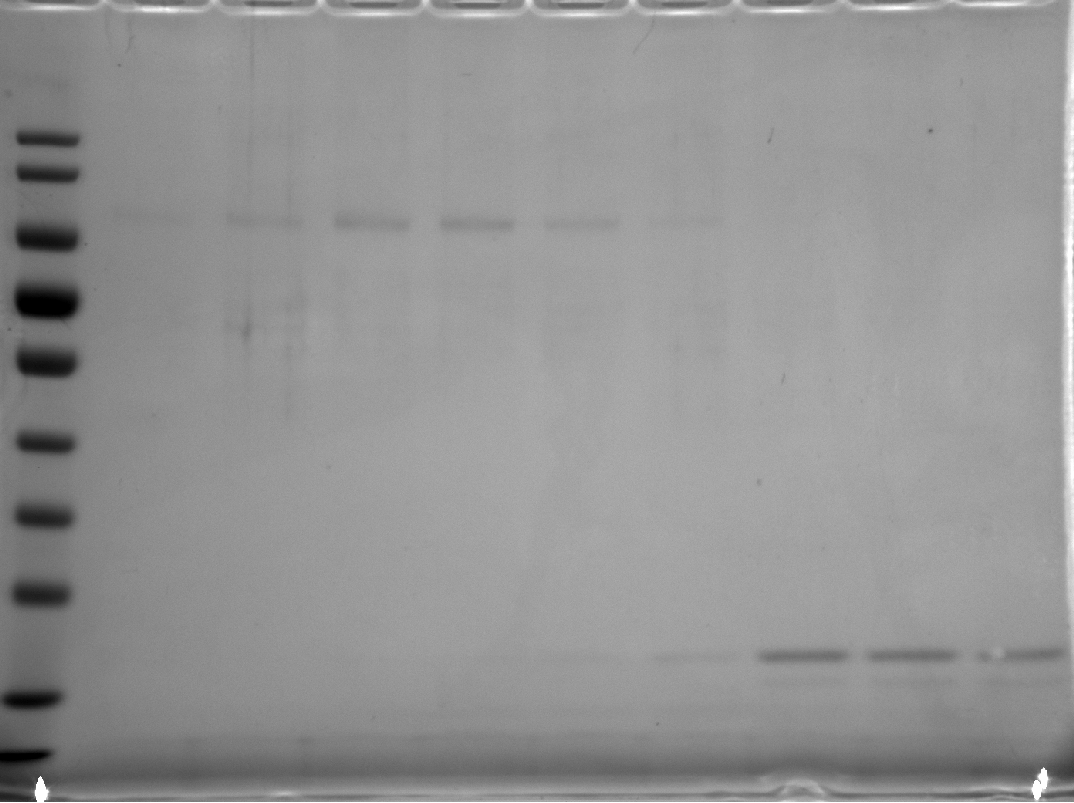

Supplement: Supplementary file 7 — EV/Appendix Source Data [file 44318_2025_373_MOESM7_ESM.zip › EMBOJ-2024-118663_SD_EV_AppendixFigures/SD_FigureEV1E_3.tif]

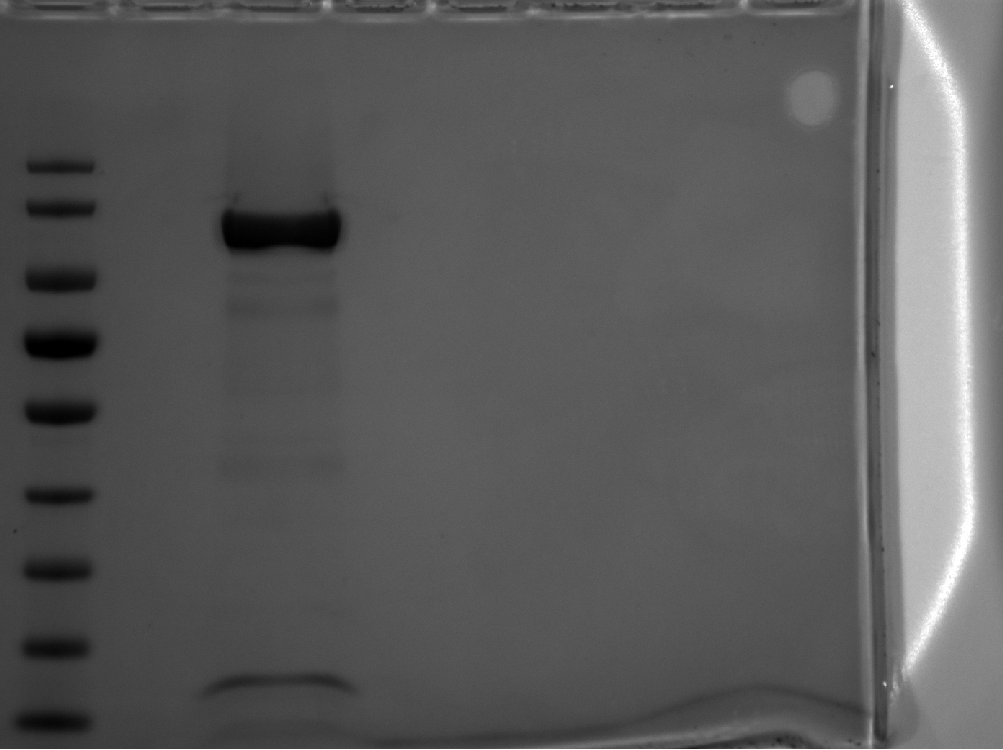

Supplement: Supplementary file 7 — EV/Appendix Source Data [file 44318_2025_373_MOESM7_ESM.zip › EMBOJ-2024-118663_SD_EV_AppendixFigures/SD_Appendix2B.tif]

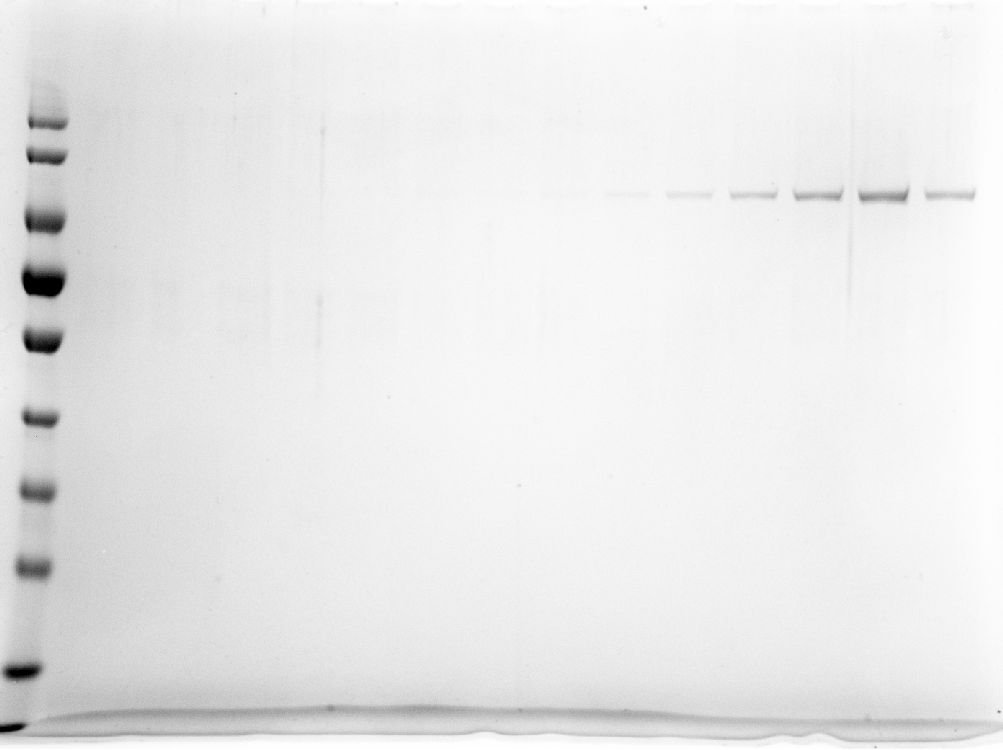

Supplement: Supplementary file 7 — EV/Appendix Source Data [file 44318_2025_373_MOESM7_ESM.zip › EMBOJ-2024-118663_SD_EV_AppendixFigures/SD_FigureEV1A_3.tif]

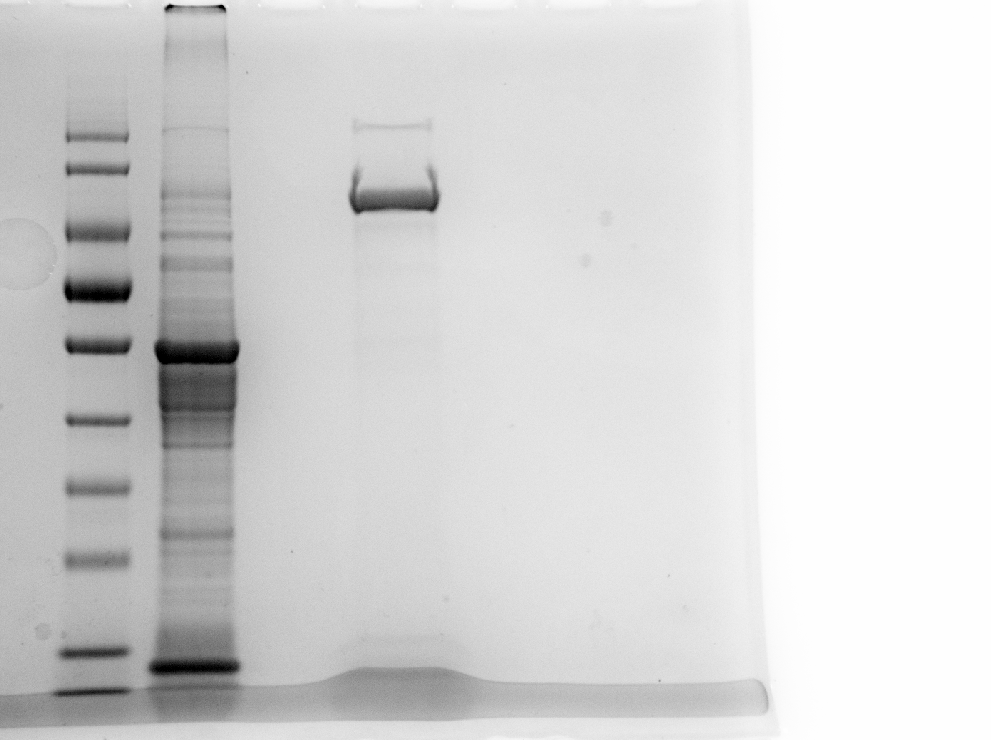

Supplement: Supplementary file 7 — EV/Appendix Source Data [file 44318_2025_373_MOESM7_ESM.zip › EMBOJ-2024-118663_SD_EV_AppendixFigures/SD_FigureEV1A_2.tif]

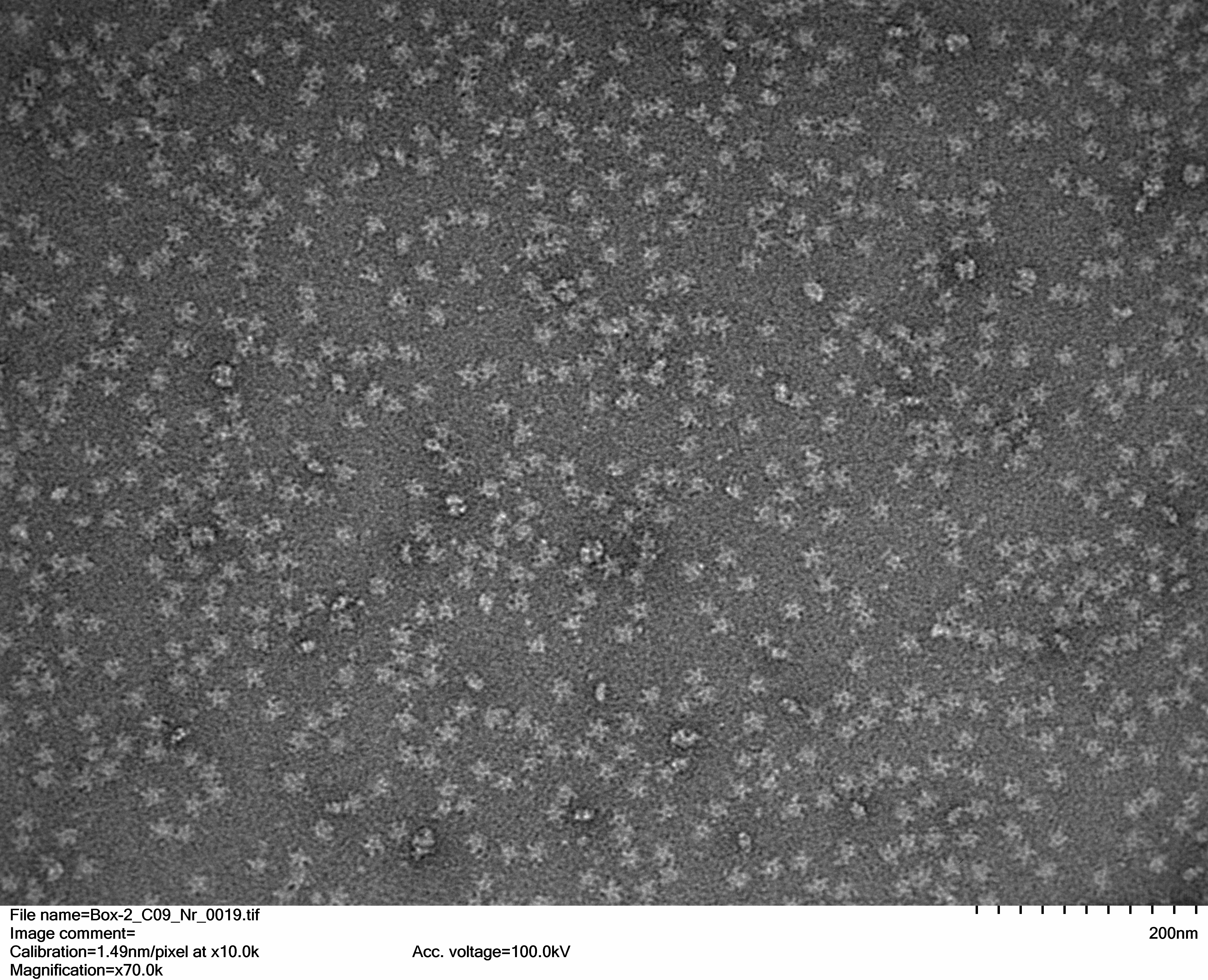

Supplement: Supplementary file 7 — EV/Appendix Source Data [file 44318_2025_373_MOESM7_ESM.zip › EMBOJ-2024-118663_SD_EV_AppendixFigures/SD_Appendix3C.tif]

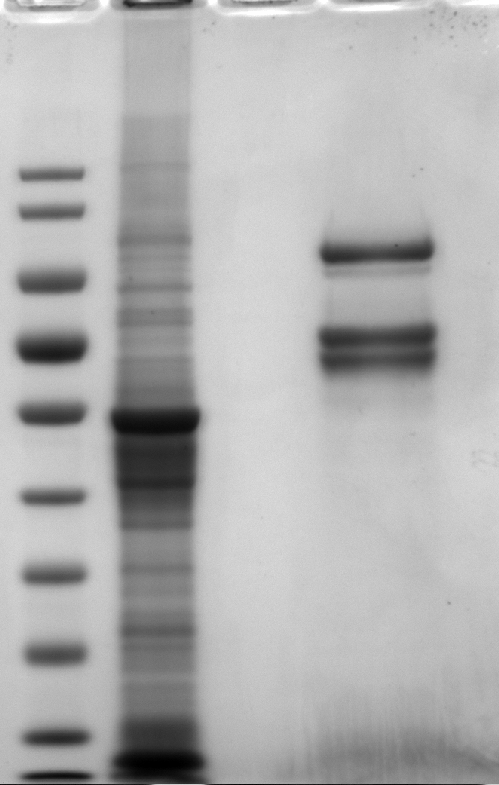

Supplement: Supplementary file 7 — EV/Appendix Source Data [file 44318_2025_373_MOESM7_ESM.zip › EMBOJ-2024-118663_SD_EV_AppendixFigures/SD_Appendix3A.tif]

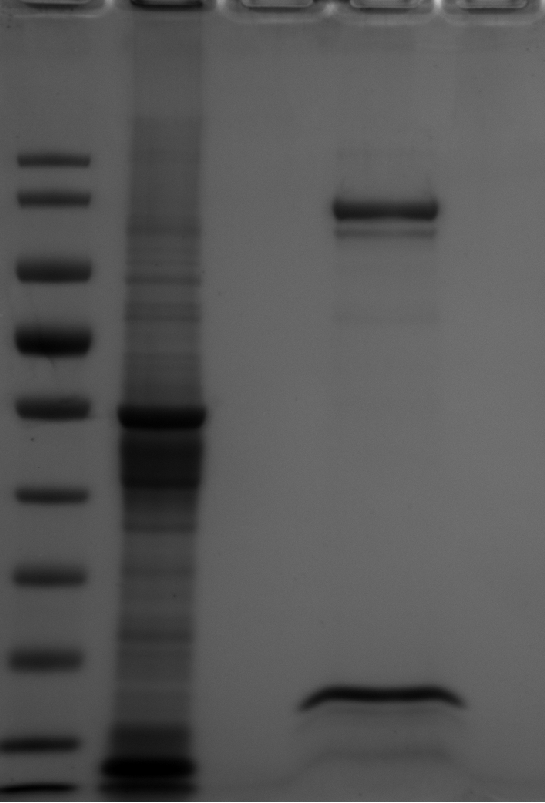

Supplement: Supplementary file 7 — EV/Appendix Source Data [file 44318_2025_373_MOESM7_ESM.zip › EMBOJ-2024-118663_SD_EV_AppendixFigures/SD_FigureEV1C_2.tif]

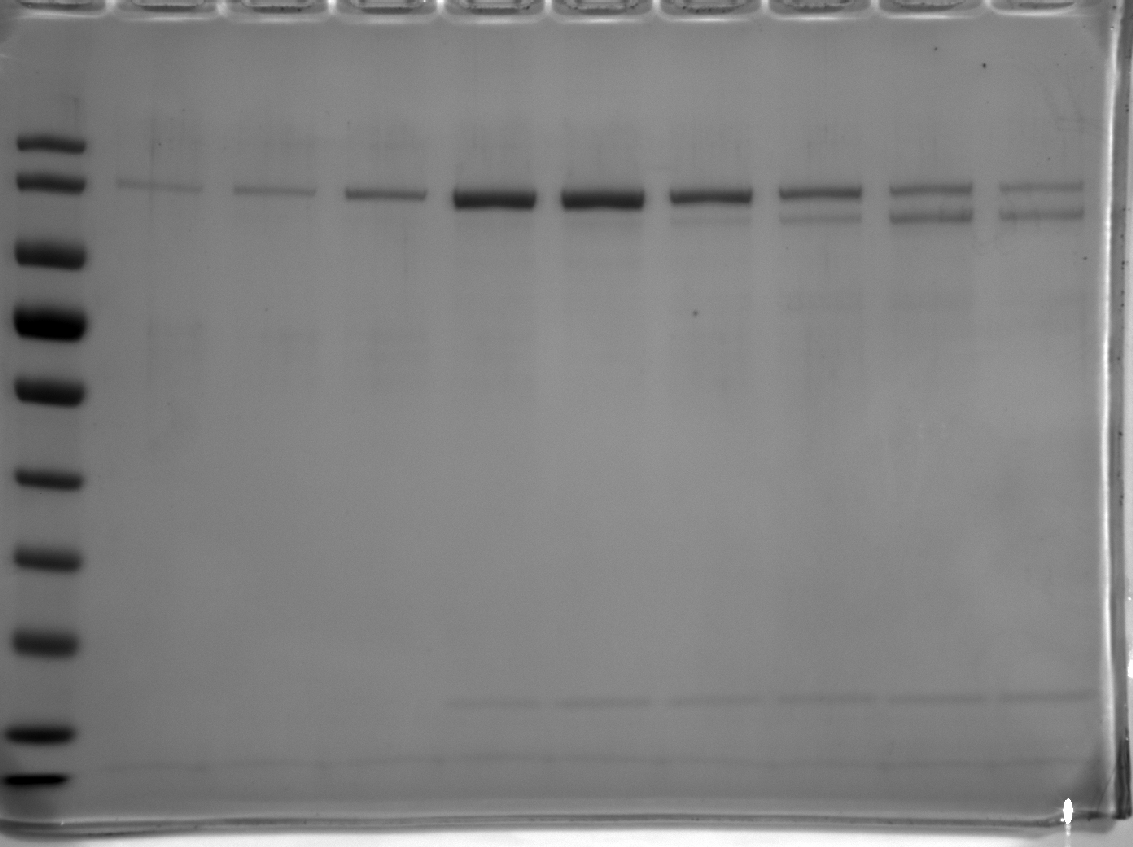

Supplement: Supplementary file 7 — EV/Appendix Source Data [file 44318_2025_373_MOESM7_ESM.zip › EMBOJ-2024-118663_SD_EV_AppendixFigures/SD_FigureEV1C_3.tif]

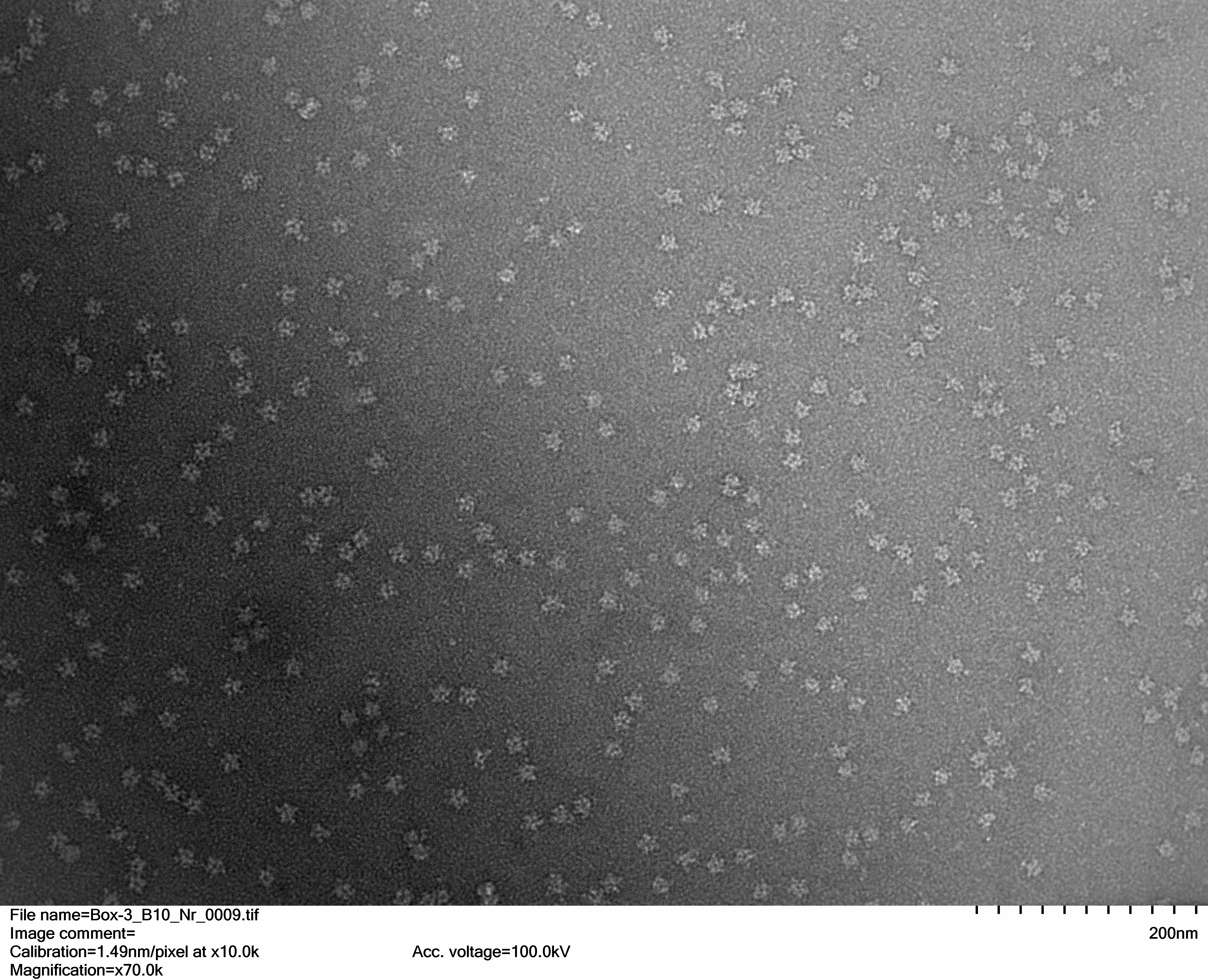

Supplement: Supplementary file 7 — EV/Appendix Source Data [file 44318_2025_373_MOESM7_ESM.zip › EMBOJ-2024-118663_SD_EV_AppendixFigures/SD_Appendix4C.tif]

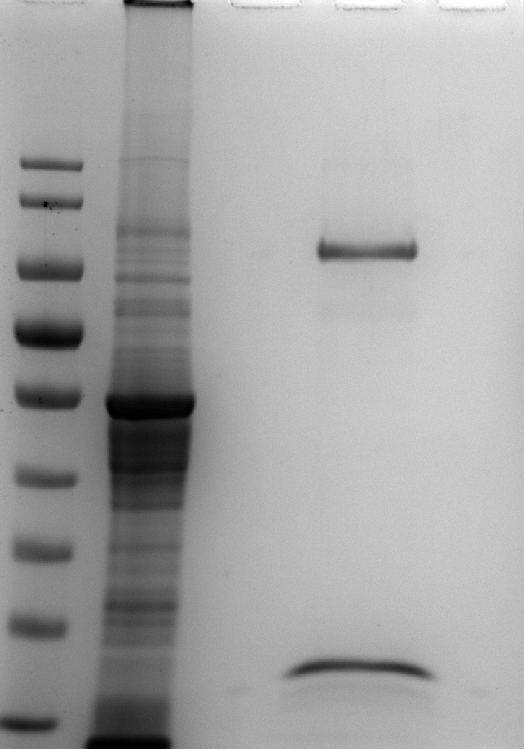

Supplement: Supplementary file 7 — EV/Appendix Source Data [file 44318_2025_373_MOESM7_ESM.zip › EMBOJ-2024-118663_SD_EV_AppendixFigures/SD_FigureEV1D_2.tif]

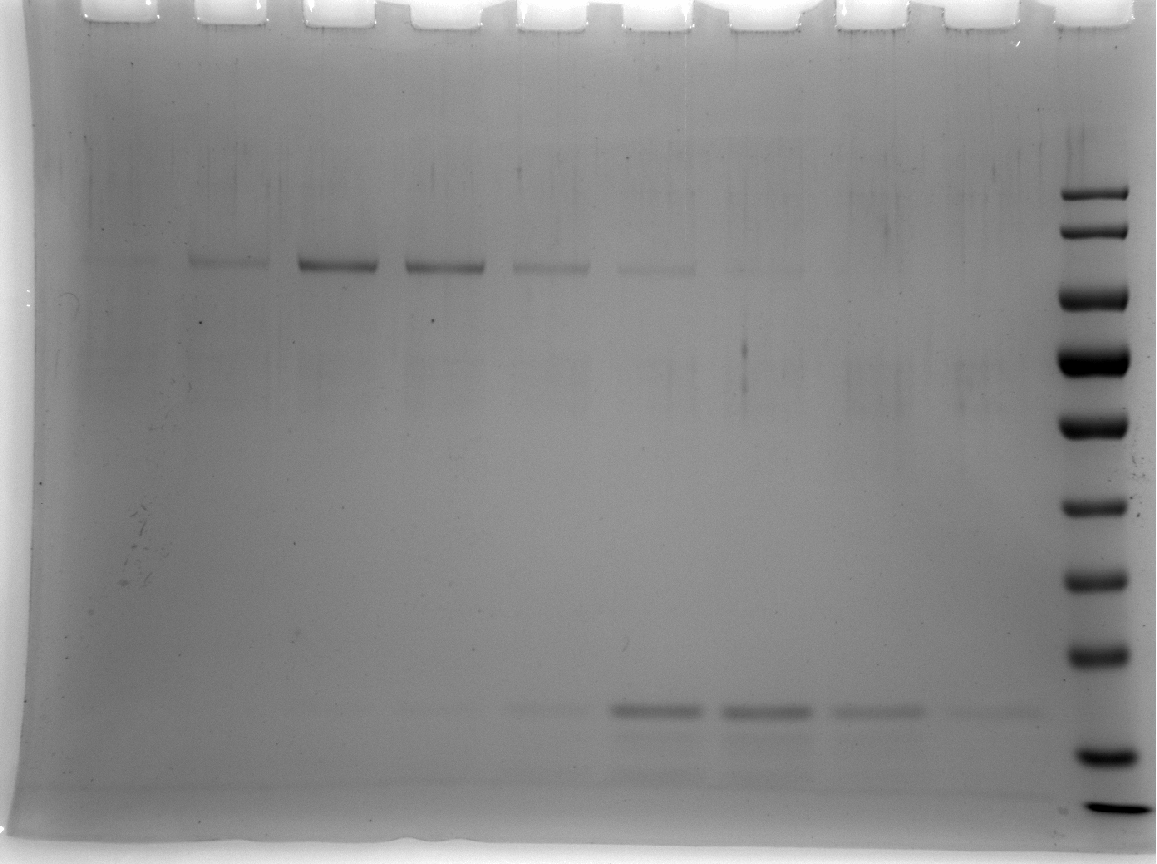

Supplement: Supplementary file 7 — EV/Appendix Source Data [file 44318_2025_373_MOESM7_ESM.zip › EMBOJ-2024-118663_SD_EV_AppendixFigures/SD_FigureEV1D_3.tif]

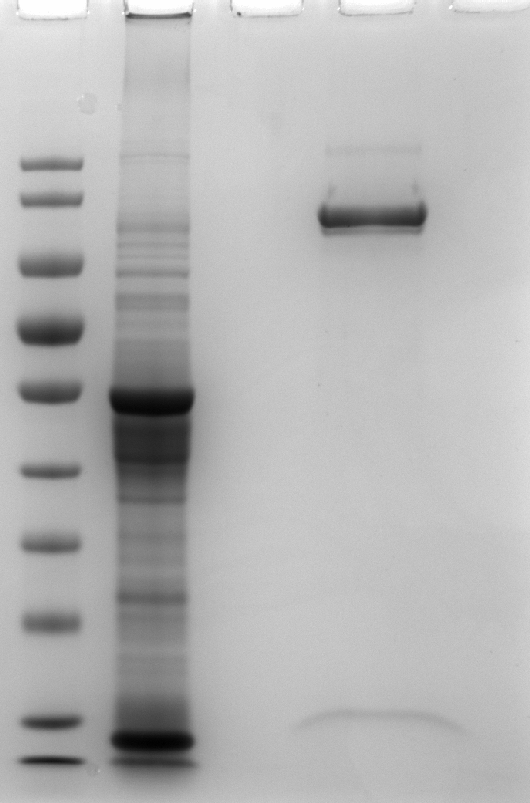

Supplement: Supplementary file 7 — EV/Appendix Source Data [file 44318_2025_373_MOESM7_ESM.zip › EMBOJ-2024-118663_SD_EV_AppendixFigures/SD_Appendix4A.tif]

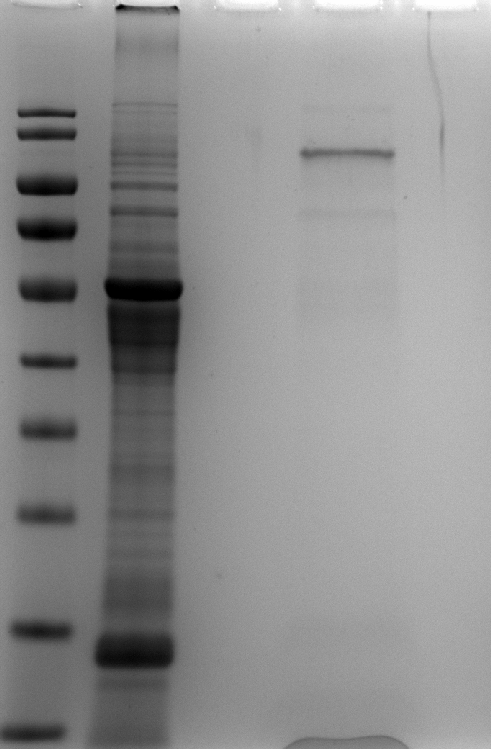

Supplement: Supplementary file 7 — EV/Appendix Source Data [file 44318_2025_373_MOESM7_ESM.zip › EMBOJ-2024-118663_SD_EV_AppendixFigures/SD_FigureEV1B_2.tif]

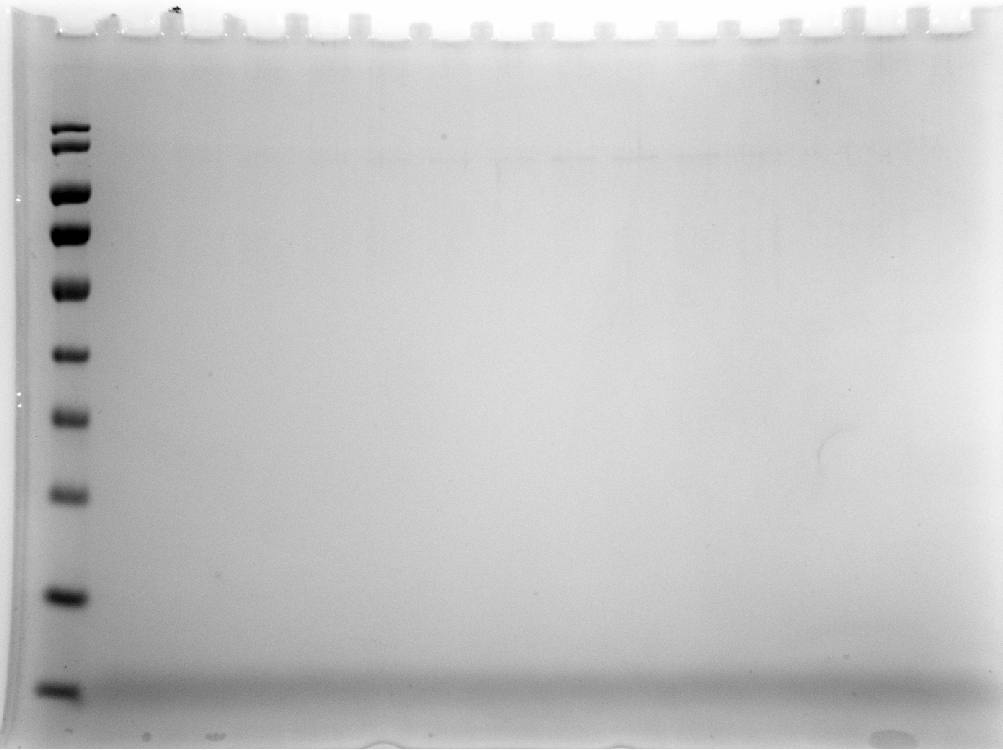

Supplement: Supplementary file 7 — EV/Appendix Source Data [file 44318_2025_373_MOESM7_ESM.zip › EMBOJ-2024-118663_SD_EV_AppendixFigures/SD_FigureEV1B_3.tif]
