# Supplementary material for: The barley MLA13-AVRA13 heterodimer reveals principles for immunoreceptor recognition of RNase-like powdery mildew effectors
Source: EMBO J. 2025 Feb 13;44(11):3210–30. doi: 10.1038/s44318-025-00373-9 (PMC12130304; doi:10.1038/s44318-025-00373-9)
Supplement: Supplementary file 8 — Expanded View Figures [file 44318_2025_373_MOESM8_ESM.pdf]

Expanded View Figures

Figure EV1. Co-expression of MLA13–AVR$_{A13}$-1 without an N-terminal GST tag on MLA13 or with various MLA13 substitutions consistently elute at a volume indicating a low-molecular-weight complex.

Elution volumes of the Sr35 resistosome, Sr50 resistosome and MLA13$^{K98E/K100E}$-AVR$_{A13}$-1 heterodimer are shown with a dotted line when purified using the same method (Appendix Figs. S3, S4 and main Fig. 1A, respectively). (**A**) MLA13$^{K98E/K100E}$-2S-HA expressed and purified alone. (**B**) MLA13$^{K98E/K100E/D502V}$-2S-HA expressed and purified alone. (**C**) MLA13$^{K98E/K100E}$-GST expressed with AVR$_{A13}$-2S-HA. (**D**) MLA13$^{K98E/K100E}$ expressed with AVR$_{A13}$-2S-HA. (**E**) MLA13$^{L11E/L15E}$ expressed with AVR$_{A13}$-2S-HA. All samples were purified from 100 g of leaf tissue and with a single-step affinity purification via the Twin-Strep-tag® (2S-HA), followed by SEC. Fractions from the SEC profiles are numbered and presented on the accompanying CBB-stained, 10 or 12% SDS-PAGE gels. Source data are available online for this figure.

© The Author(s)

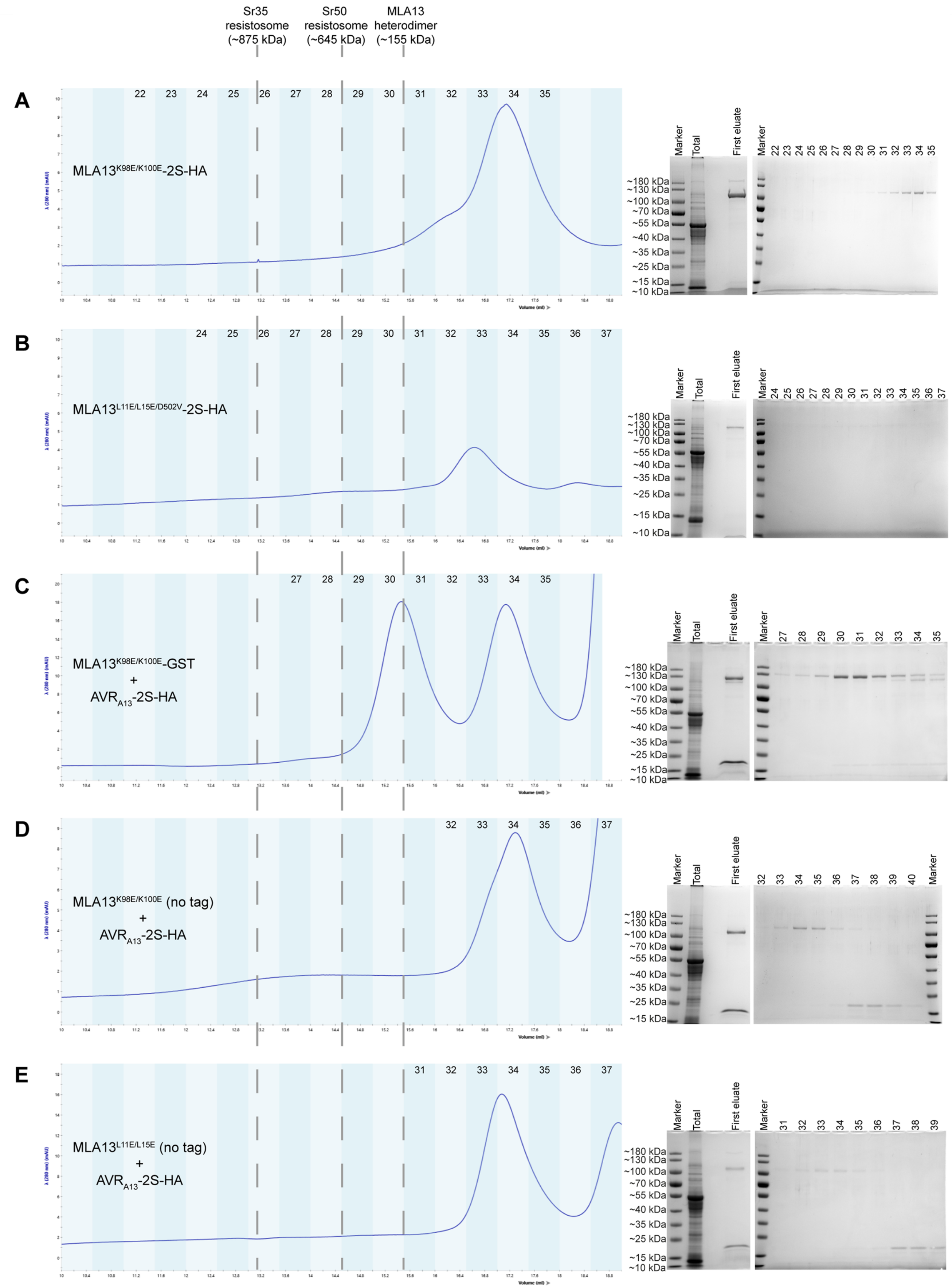
Sr35 resistosome (~875 kDa)
Sr50 resistosome (~645 kDa)
MLA13 heterodimer (~155 kDa)
A
MLA13K98E/K100E-2S-HA
B
MLA13L11E/L15E/D502V-2S-HA
C
MLA13K98E/K100E-GST + AVRA13-2S-HA
D
MLA13K98E/K100E (no tag) + AVRA13-2S-HA
E
MLA13L11E/L15E (no tag) + AVRA13-2S-HA
Marker
Total
First eluate
~180 kDa
~130 kDa
~100 kDa
~70 kDa
~55 kDa
~40 kDa
~35 kDa
~25 kDa
~15 kDa
~10 kDa
λ (280 nm) (mAU)
Volume (ml)

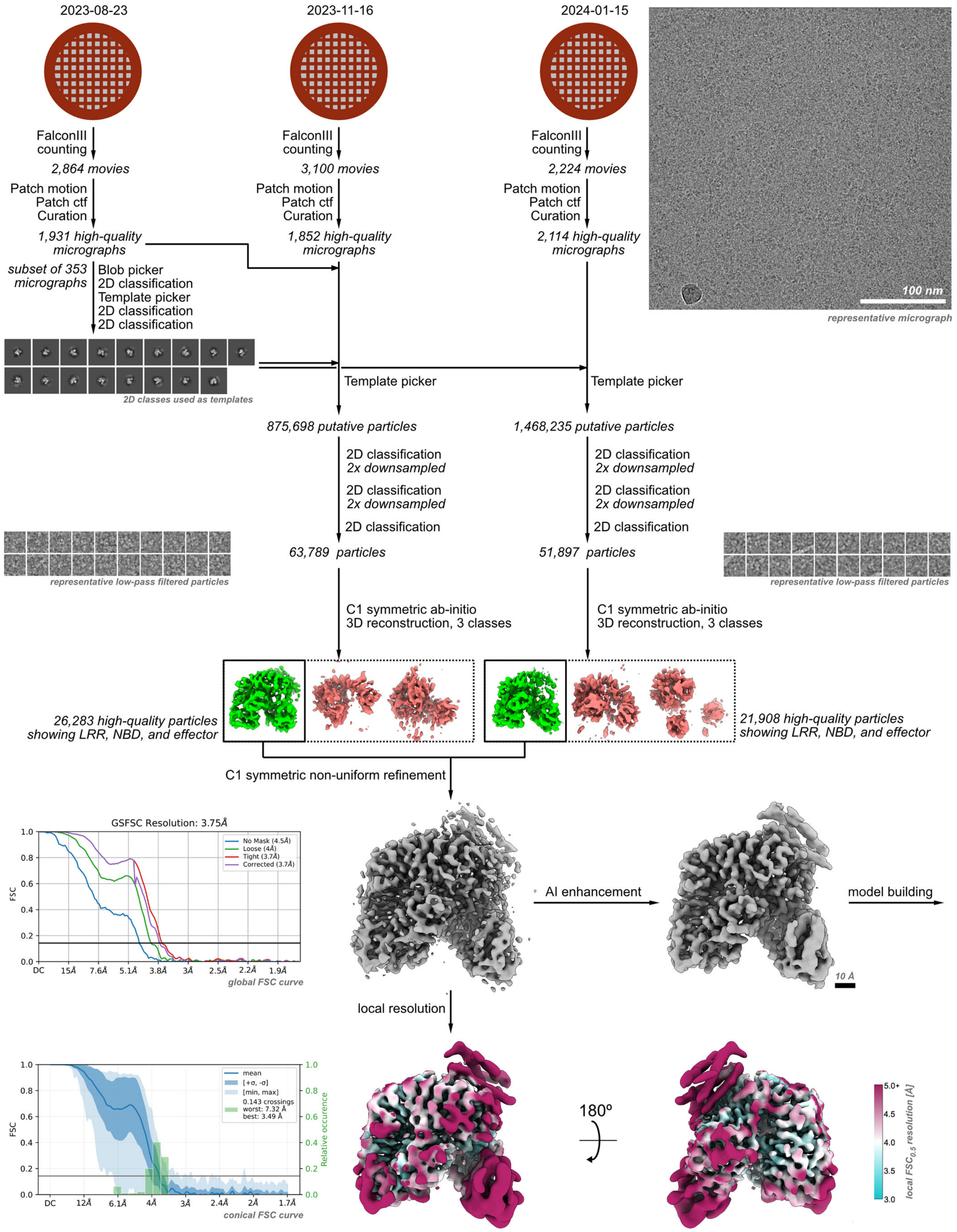
2023-08-23
2023-11-16
2024-01-15
FalconIII counting
2,864 movies
3,100 movies
2,224 movies
Patch motion
Patch ctf
Curation
1,931 high-quality micrographs
1,852 high-quality micrographs
2,114 high-quality micrographs
subset of 353 micrographs
Blob picker
2D classification
Template picker
2D classification
2D classification
100 nm
representative micrograph
2D classes used as templates
Template picker
875,698 putative particles
1,468,235 putative particles
2D classification
2x downsampled
63,789 particles
51,897 particles
representative low-pass filtered particles
C1 symmetric ab-initio
3D reconstruction, 3 classes
26,283 high-quality particles showing LRR, NBD, and effector
21,908 high-quality particles showing LRR, NBD, and effector
C1 symmetric non-uniform refinement
GSFSC Resolution: 3.75Å
No Mask (4.5Å)
Loose (4Å)
Tight (3.7Å)
Corrected (3.7Å)
FSC
global FSC curve
AI enhancement
model building
10 Å
local resolution
mean
[+σ, -σ]
[min, max]
0.143 crossings
worst: 7.32 Å
best: 3.49 Å
Relative occurence
conical FSC curve
180º
local FSC0.5 resolution [Å]

Figure EV2. Workflow of cryo-EM data acquisition and analysis of the MLA13$^{K98E/K100E}$-AVR$_{A13}$-1 heterodimer.

A total of three datasets were collected on a 300 kV cryo-electron microscope. For each dataset, movies were selected for low per-frame drift rates, good CTF scores, and low astigmatism. Particles were first picked using a blob picker, and then subjected to unsupervised 2D classification. Representative classes showing protein-like structures were used for a template picker. Putative detected particles were curated using unsupervised 2D classification, selecting for particles with protein-like density and resolutions better than 10 Å. The selected particles were further curated using ab initio reconstruction, sorting them into three distinct populations. From these, all particles contributing to a structure showing clear density for the LRR, NBD and effector (shown in green and highlighted by a thicker box outline) were combined and refined in 3D using a non-uniform refinement algorithm, resulting in a map with a global resolution of 3.8 Å. Before model building, the map was further sharpened using DeepEMhancer. Source data are available online for this figure.

 © The Author(s)

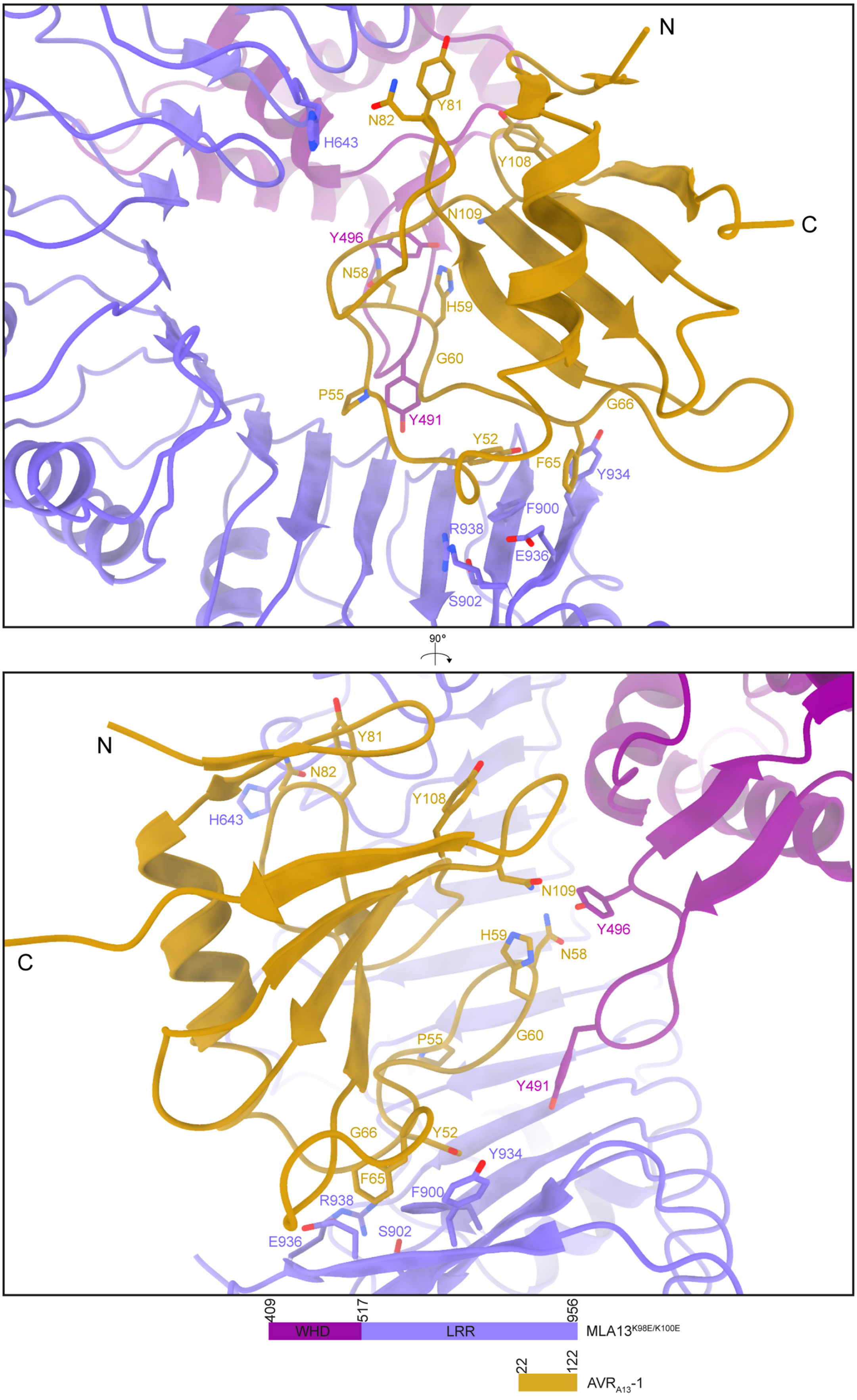
N
C
Y81
N82
H643
Y108
N109
Y496
N58
H59
G60
P55
Y491
G66
Y52
F65
Y934
F900
R938
E936
S902
90°
409
517
956
WHD
LRR
MLA13K98E/K100E
22
122
AVRA13-1

Figure EV3. The MLA13$^{K98E/K100E}$-AVR$_{A13}$-1 interface from two different angles.

Atomic model showing residues predicted to contribute to the interface and/or experimentally tested for loss of MLA13-mediated cell death. Source data are available online for this figure.

 © The Author(s)

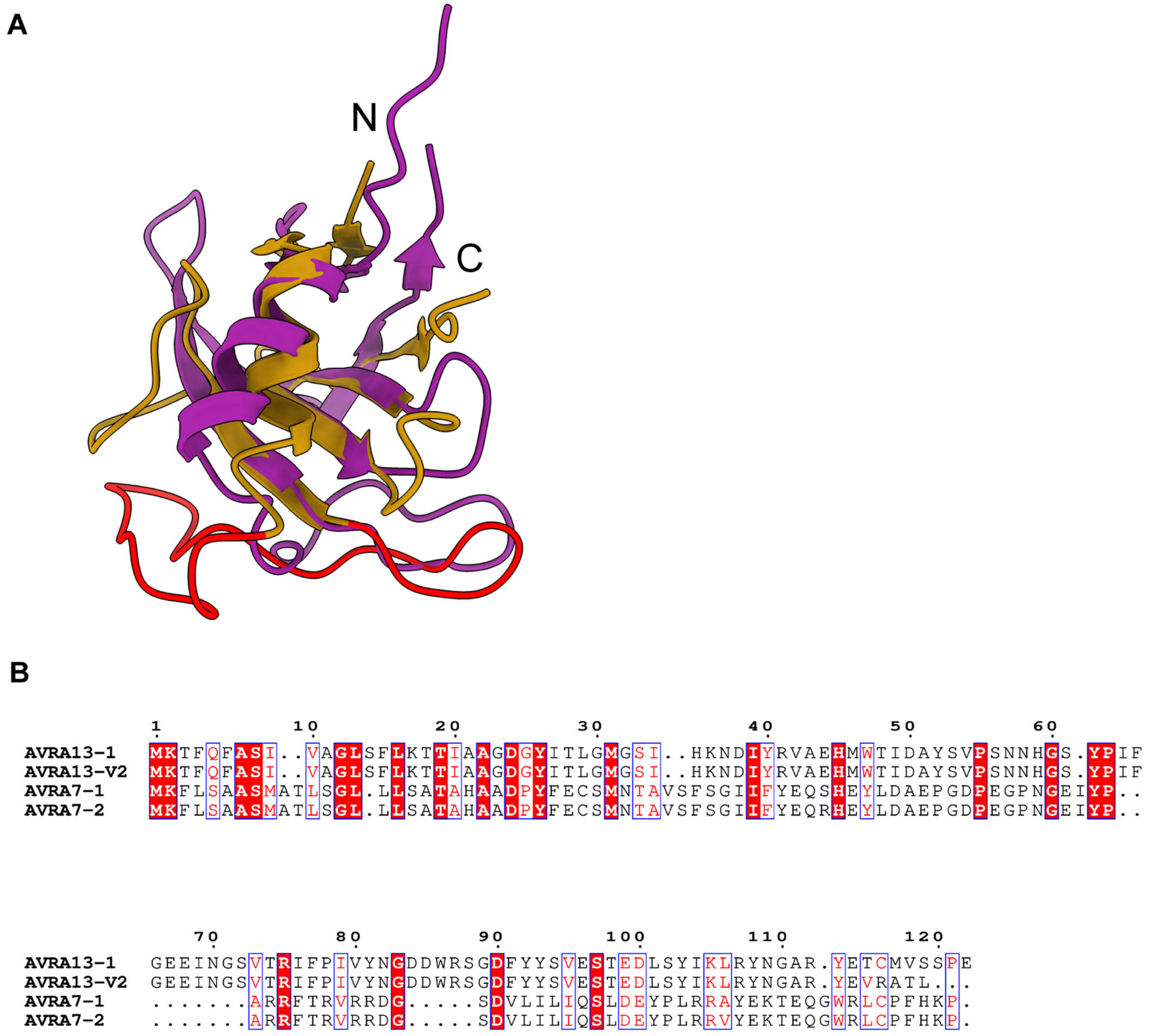

Figure EV4. Structural and sequence alignments of AVR_{A13} and AVR_{A7} variants.

(**A**) Structural alignment of AVR_{A13}-1 (dark goldenrod colour) and crystal structure of AVR_{A7}-1 (burgundy colour; PDB: 8OXL). The basal loops of AVR_{A13}-1 are coloured in red. (**B**) Sequence alignment of AVR_{A13} and AVR_{A7} variants. Alignment performed using MUSCLE and visualised using ESPript 3.0. Source data are available online for this figure.

© The Author(s)
